# The role of nutritional vitamin D in chronic kidney disease–mineral and bone disorder in children and adults with chronic kidney disease, on dialysis, and after kidney transplantation—a European consensus statement

**DOI:** 10.1093/ndt/gfae293

**Published:** 2025-01-28

**Authors:** Hanne Skou Jørgensen, Marc Vervloet, Etienne Cavalier, Justine Bacchetta, Martin H de Borst, Jordi Bover, Mario Cozzolino, Ana Carina Ferreira, Ditte Hansen, Markus Herrmann, Renate de Jongh, Sandro Mazzaferro, Mandy Wan, Rukshana Shroff, Pieter Evenepoel

**Affiliations:** Department of Clinical Medicine, Aarhus University, Aarhus, Denmark; Department of Nephrology, Aalborg University Hospital, Aalborg, Denmark; Department of Nephrology, Radboud University Medical Center, Nijmegen, The Netherlands; Department of Clinical Chemistry, CIRM, University of Liege, CHU de Liège, Liège, Belgium; Department of Pediatric Nephrology, Reference Center for Rare Diseases of Calcium and Phosphate, INSERM1033 Research Unit, Hospices Civils de Lyon, Université Lyon 1, Lyon, France; Department of Internal Medicine, Division of Nephrology, University Medical Center Groningen, University of Groningen, Groningen, the Netherlands; Department of Nephrology, University Hospital Germans Trias i Pujol, Badalona (Barcelona), Catalonià, Spain; Department of Health Sciences, Renal Division, University of Milan, Milan, Italy; Nephrology Department, Hospital Curry Cabral | ULS São José, Lisbon, Portugal and Nova Medical School, Lisbon, Portugal; Department of Nephrology, Copenhagen University Hospital-Herlev, Copenhagen, Denmark; Department of Clinical Medicine, University of Copenhagen, Copenhagen, Denmark; Clinical Institute of Medical and Chemical Laboratory Diagnostics, Medical University of Graz, Graz, Austria; Department of Endocrinology and Metabolism, Amsterdam UMC Location Vrije Universiteit Amsterdam, Amsterdam, The Netherlands; Department of Translation and Precision Medicine, Sapienza University of Rome, Rome, Italy; Institute of Pharmaceutical Science, King's College London, London, UK and Department of Evelina Pharmacy, Guys' & St Thomas' NHS Foundation Trust, London, UK; Renal Unit, UCL Great Ormond Street Hospital for Children; University College London, London, UK; Department of Microbiology, Immunology and Transplantation; Nephrology and Renal Transplantation Research Group, KU Leuven, Leuven, Belgium

**Keywords:** chronic kidney disease–mineral and bone disorder, kidney transplantation, parathyroid hormone, renal osteodystrophy, vitamin D

## Abstract

Vitamin D deficiency is common in patients with chronic kidney disease (CKD) and associates with poor outcomes. Current clinical practice guidelines recommend supplementation with nutritional vitamin D as for the general population. However, recent large-scale clinical trials in the general population failed to demonstrate a benefit of vitamin D supplementation on skeletal or non-skeletal outcomes, fueling a debate on the rationale for screening for and correcting vitamin D deficiency, both in non-CKD and CKD populations. In a collaboration between the European Renal Osteodystrophy initiative of the European Renal Association (ERA) and the European Society for Paediatric Nephrology (ESPN), an expert panel performed an extensive literature review and formulated clinical practice points on vitamin D supplementation in children and adults with CKD and after kidney transplantation. These were reviewed by a Delphi panel of members from relevant working groups of the ERA and ESPN. Key clinical practice points include recommendations to monitor for, and correct, vitamin D deficiency in children and adults with CKD and after kidney transplantation, targeting 25-hydroxyvitamin D levels >75 nmol/l (>30 ng/ml). Although vitamin D supplementation appears well-tolerated and safe, it is recommended to avoid mega-doses (≥100 000 IU) and very high levels of 25 hydroxyvitamin D (>150–200 nmol/l, or 60–80 ng/ml) to reduce the risk of toxicity. Future clinical trials should investigate the benefit of vitamin D supplementation on patient-relevant outcomes in the setting of vitamin D deficiency across different stages of CKD.

## Graphical Abstract

**Graphical Abstract:**
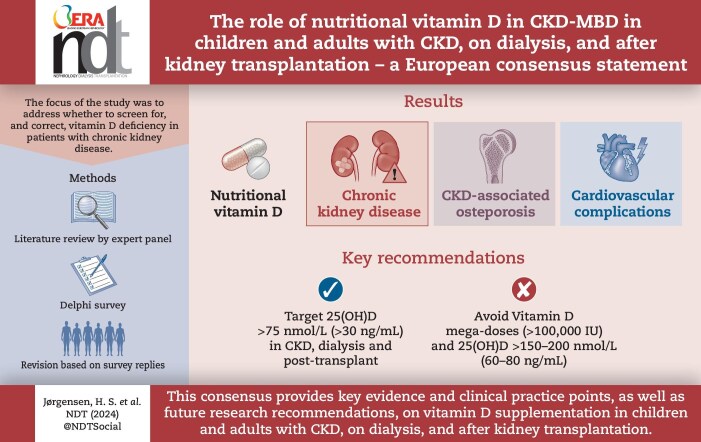

## INTRODUCTION

Vitamin D deficiency is common in patients with chronic kidney disease (CKD) and associates with poor outcomes. Even so, the evaluation and management of vitamin D deficiency in patients with CKD remains controversial. Therapy with active vitamin D compounds in patients with CKD failed to improve outcomes and is known to confer risk of hypercalcemia [1–3]. Current clinical practice guidelines state that it is reasonable to reserve the use of calcitriol and active vitamin D analogues for patients with advanced CKD and severe and progressive hyperparathyroidism [4, 5]. Nutritional vitamin D supplementation is recommended as for the general population [4, 5].

Recently, large randomized controlled trials (RCTs), such as the vitamin D and omega-3 Trial (VITAL), failed to show health benefits of nutritional vitamin D supplementation in the general population, even in individuals with low 25-hydroxy vitamin D [25(OH)D] levels at baseline [6, 7]. Following the publication of these trials, key opinion leaders suggested that healthcare providers should stop screening for vitamin D deficiency [8], and that people should stop taking vitamin D supplements [9]. Others advocated for a more nuanced interpretation [10–12]. The results of these trials are diffusing into the nephrology community [13], raising the question of whether nutritional vitamin D supplementation should also be abandoned in patients with CKD.

Besides this ‘existential question’ on the need for screening for and correcting vitamin D deficiency, other questions related to the optimal monitoring [14] (quantitative measures versus vitamin D metabolites ratios, total versuss free 25(OH)D) and treatment strategy (target 25(OH)D level, combination therapy of nutritional vitamin D and active vitamin D compounds) in patients with CKD should be considered as well [15]. This consensus statement addresses these questions and aims to provide guidance on how to monitor for and correct vitamin D deficiency using nutritional vitamin D supplements in children and adults with CKD, on dialysis, and after kidney transplantation.

## METHODS

### Developing the PICO questions

We developed clinical questions to be addressed and framed them in a searchable format, with specification of the population (P) to whom the statements would be applicable; the intervention (I) considered; the comparator (C; which may be placebo, ‘no therapy’ or alternative intervention); and the outcomes (O) of interest. Our PICO terms were as follows:

**Population:** All patients (children and adults) with CKD grades 2–5, on dialysis, and after kidney transplantation

**Intervention:** Nutritional vitamin D supplementation (cholecalciferol, ergocalciferol, calcifediol)

**Comparator:** Placebo or no supplementation, standard of care, active vitamin D compounds

**Outcomes:**

- Patient-level outcomes: All-cause mortality, cardiovascular mortality, major adverse cardiovascular events (MACE), falls in the elderly, fractures, growth in children.
- Bone imaging: Bone mineral density (BMD)
- Bone histology: Measures of bone turnover, mineralization, and volume
- Surrogate measures of cardiovascular disease: vascular stiffness, vascular calcification, endothelial dysfunction and blood pressure
- Biomarkers: Parathyroid hormone (PTH), serum calcium (for toxicity), fibroblast growth factor 23 (FGF23), total alkaline phosphatase and bone turnover markers (particularly non-renally excreted markers such as bone-specific alkaline phosphatase, BALP), 25(OH)D and its metabolites, vitamin D metabolic ratio(s)

Outcomes outside the scope of this review include the potential effects of vitamin D supplementation on progression of CKD and albuminuria, infection risk, malignancies, inflammation, autoimmune diseases, and others.

### Literature search

A comprehensive literature search was conducted with the aim of summarizing available evidence based on the following hierarchy of studies; systematic meta-analysis, trials with clinical outcomes, trials with intermediate outcomes, prospective cohort studies with clinical outcomes, and cross-sectional studies with intermediate outcomes when a higher level of evidence was not available. Existing guidelines on recommended daily intake and target ranges of vitamin D for the general population were reviewed. In the absence of applicable studies, guidance is based on expert opinion.

### Framing advice

A summary of the available evidence from the literature is presented as ‘Key evidence points’, whereas ‘Clinical practice points’ represent the resulting clinical practice recommendations. The final draft of the manuscript was sent to members of the CKD–mineral and bone disorder (CKD-MBD) working group of the European Renal Association and the CKD-MBD and Dialysis working groups of the European Society for Paediatric Nephrology (ESPN) with an e-questionnaire to provide a level of agreement on a 5-point scale (strongly disagree, disagree, neither agree nor disagree, agree, strongly agree) for the practice points. Participants were also given the opportunity of providing direct feedback, including suggestions for rewording of the practice points. A priori, an agreement of >70% was required for each statement. If this condition was not fulfilled, the practice point would be discussed and adapted by the writing team.

## Q1: What are the current recommendations for vitamin D supplementation in the general population?

### 1.1 Vitamin D metabolism in health

#### Key evidence points

- Dermal production upon ultraviolet-B light exposure is the most important source of vitamin D in humans as most foods have a low natural content of vitamin D.
- Liver 25-hydroxylase activity is metabolically controlled, rather than constitutively determined.
- Renal 1,25 dihydroxy vitamin D (1,25(OH)_2_D) production and degradation is tightly controlled by the hormones that govern mineral metabolism (PTH, FGF23, vitamin D metabolites).
- Extrarenal 1,25(OH)_2_D production is largely substrate-dependent and may assist in maintaining circulating 1,25(OH)_2_D levels, at least in conditions of insufficient renal production.

#### Background and rationale

**Figure 1:**
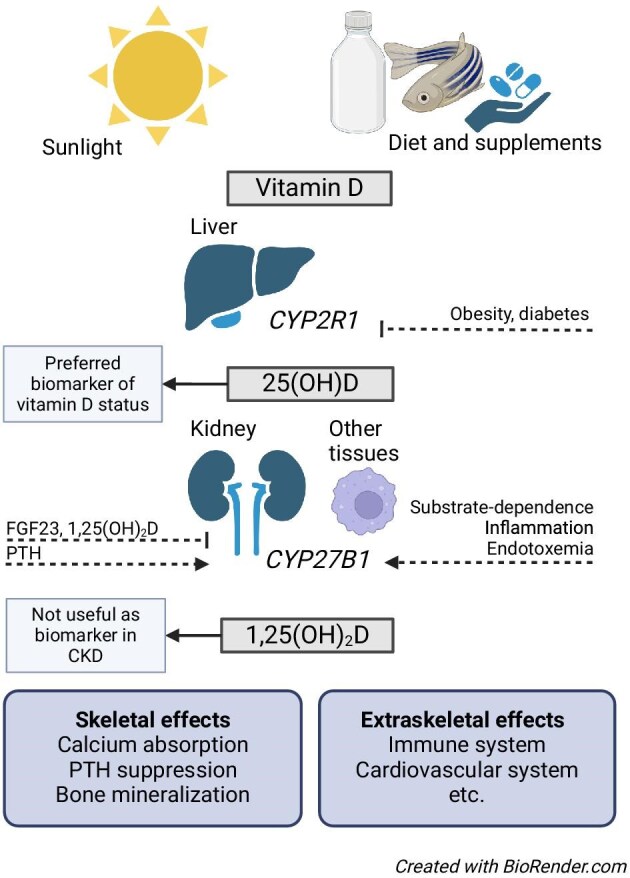
Overview of vitamin D metabolism. Vitamin D is supplied through ultraviolet radiation of the skin or through diet. Two hydroxylation steps are necessary for activation into the active hormone, first by *CYP2R1* in the liver to 25 hydroxyvitamin D [25(OH)D], and then by *CYP27B1* in the kidneys or other tissues to 1,25 dihydroxy vitamin D [1,25(OH)2D]. Hepatic hydroxylation may be affected by energy metabolism, with reduced efficacy seen in diabetes mellitus and obesity. Renal *CYP27B1* is under strict hormonal control by hormones governing mineral metabolism (parathyroid hormone, PTH and fibroblast growth factor 23, FGF23), with negative feedback from 1,25(OH)2D itself. In contrast, extrarenal *CYP27B1* activity seems to be substrate dependent and stimulated by conditions of inflammation.

Vitamin D is not a single compound but refers to a group of over 50 metabolites. Cholecalciferol (vitamin D_3_) and ergocalciferol (vitamin D_2_) are the two parent forms of vitamin D. The dietary intake of vitamin D is limited, as most foods (apart from oily fish [16]) have a low natural content of vitamin D. In most countries, the average daily intake of vitamin D is <5 µg (1 µg = 40 IU), and even in countries where foods are enriched with vitamin D, the total vitamin D intake is often lower than 10 µg/day [17]. Thus, humans are largely dependent on ultraviolet-B radiation-induced cutaneous synthesis of vitamin D_3_ (cholecalciferol) [16]. Vitamin D is inactive and requires several metabolic steps, first by hydroxylation in the liver (mostly, but not exclusively, by *CYP2R1*, 25-hydroxylase) into 25(OH)D, followed by a second hydroxylation by *CYP27B1* (1-α-hydroxylase) into 1,25(OH)_2_D to become fully active (Fig. 1) [18]. *CYP24A1* is the main enzyme responsible for the catabolism of all vitamin D metabolites. Through a multistep pathway, this catabolism results in a large number of mostly inactive metabolites with side-chain modifications, ultimately ending with calcitroic and calcioic acid.

**Figure 2:**
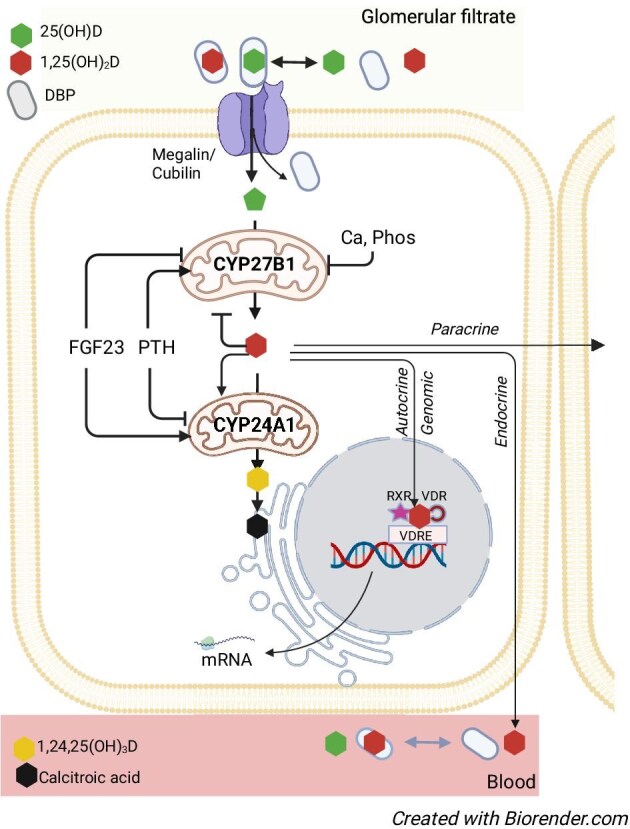
Renal vitamin D metabolism and primary regulators of 1α-hydroxylase and 24-hydroxylase enzymes. Vitamin D metabolites circulate bound to proteins, mainly vitamin D protein. The 25-hydroxyvitamin D-DBP complex enters the proximal tubular cells from the glomerular filtrate through a megalin/cubulin-mediated transport. 1α-Hydroxylase (encoded by *CYP27B1*) is a cytochrome P450 enzyme that catalyzes the hydroxylation of 25-hydroxyvitamin D [25(OH)D] to 1,25-dihydroxyvitamin D [1,25(OH)2D; the active form of vitamin D]. 24-hydroxylase (encoded by *CYP24A1*) catalyzes the 24-hydroxylation of 25(OH)D and 1,25(OH)2D to their inactive 24-metabolites. Factors that regulate each enzyme are depicted in the figure. Renally produced 1,25(OH)2D serves autocrine (genomic and non-genomic), paracrine and endocrine functions. Abbreviations: FGF23, fibroblast growth factor 23; PTH, parathyroid hormone; DBP, Vitamin D binding protein; Ca, calcium; Phos, Phosphate; VDRE, vitamin D responsive element; VDR, vitamin D receptor; RXR, retinoid X receptor.

**Figure 3:**
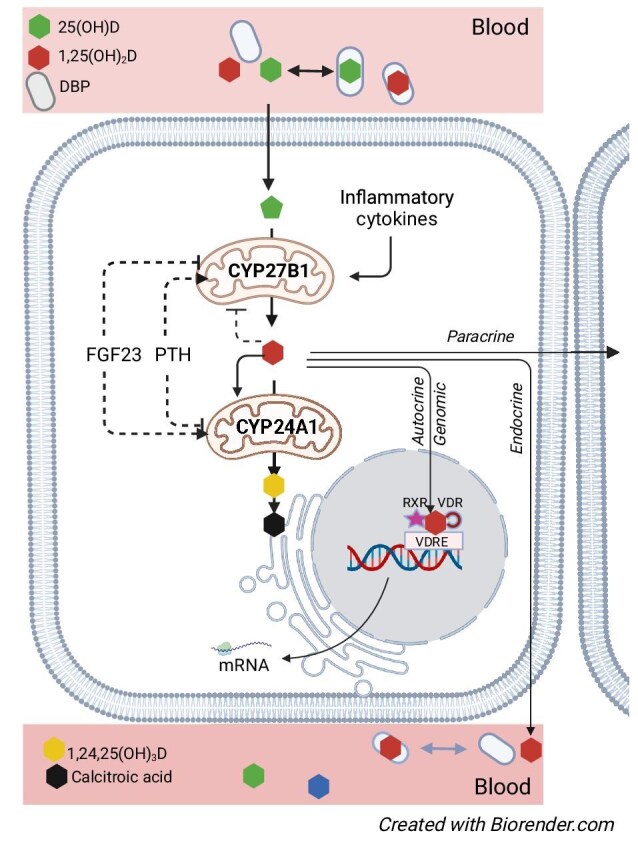
Extrarenal vitamin D metabolism and primary regulators of 1α-hydroxylase and 24-hydroxylase enzymes. Free circulating 25-hydroxyvitamin D enters the cells through diffusion. Factors that regulate extrarenal *CYP27B1* and *CYP24A1* are depicted in the figure. Extrarenally produced 1,25(OH)2D mainly serves autocrine (genomic and non-genomic) and paracrine functions, but may also spill over the circulation and thus confer endocrine actions. Abbreviations: 24,25(OH)2D, 24,25-dihydroxyvitamin D; FGF23, fibroblast growth factor 23; PTH, parathyroid hormone; DBP, Vitamin D binding protein; VDRE, vitamin D responsive element; VDR, vitamin D receptor; RXR, retinoid X receptor.

Recent findings indicate that *CYP2R1* expression is not constitutively determined, but under the control of metabolic signals induced by energy metabolism (fasting, diabetes) or exposure to high-dose glucocorticoids [19, 20]. In contrast to *CYP2R1, CYP27B1* and *CYP24A1* are widely expressed across different body tissues [21]. Renal *CYP27B1* and *CYP24A1* expression levels are transcriptionally regulated, mainly by the hormones of mineral metabolism, PTH, FGF23 [22], and 1,25(OH)_2_D. Reciprocal regulation of *CYP27B1* and *CYP24A1* in the kidney acts to raise or lower 1,25(OH)_2_D to maintain physiologically appropriate levels of the hormone (Fig. 2). In extrarenal tissues, hormonal regulation of *CYP27B1* is variable and likely tissue specific (Fig. 3) [23–26]. *CYP24A1* regulation is limited to induction by 1,25(OH)_2_D [27], which, at least in osteoblasts, may be amplified by PTH [28]. Production of 1,25(OH)_2_D in extrarenal tissues seems mainly driven by substrate availability [29] with activation/upregulation by inflammatory mediators such as lipopolysaccharides and interferon-gamma [30].

Vitamin D metabolites in serum are bound with high affinity to the specific vitamin D binding protein (DBP), and to a lesser extent to albumin. DBP is highly polymorphic and circulates in high concentrations, so that the free concentrations of all vitamin D metabolites are low. For most tissues, only the unbound metabolites can cross the cell membrane through diffusion. In the kidneys, parathyroid glands, and placenta, 25(OH)D and 1,25(OH)_2_D bound to DBP may also enter cells by endocytosis via megalin/cubilin [31]. Once internalized, 1,25(OH)_2_D binds to the vitamin D receptor (VDR), present in most cells. Vitamin D regulates a large number of genes, mainly related to calcium and phosphate metabolism, but also others that are involved in iron metabolism, the immune and cardiovascular systems, and overall regulation of cell proliferation and differentiation [32].

A prevailing paradigm in vitamin D metabolism is that the kidney is the only relevant source of circulating 1,25(OH)_2_D, whereas extrarenal *CYP27B1* activity serves only paracrine or autocrine functions [18, 33]. This paradigm is challenged by experimental data from a kidney-specific *CYP27B1* pseudo-null mouse model [34] and by clinical data in anephric patients [29, 35], which indicate that extrarenal *CYP27B1* activity may contribute to maintaining circulating 1,25(OH)_2_D levels, at least in conditions of defective renal *CYP27B1* activity (Fig. 3).

### 1.2 Nutritional vitamin D targets and supplementation guidelines in the general population

#### Key evidence points

- Infants and small children should be supplemented with vitamin D (400–600 IU/day), at least during their first years of life.
- Adults, especially elderly and pregnant individuals, should have access to vitamin D supplementation, but recommended doses vary widely (from 200 IU to 2000 IU/day), in line with the uncertainty regarding the minimal desirable serum concentration of 25(OH)D.

#### Background and rationale

Several groups of people have a shortfall between their requirement for vitamin D and the combined cutaneous synthesis and food intake and may therefore need vitamin D supplementation. Governments, scientific societies, and expert panels are regularly updating recommendations for the intake of vitamin D, especially for groups that should (infants) or prefer to (especially elderly individuals) avoid direct sunlight exposure. There is a fair consensus that all infants and small children should receive 400–600 IU daily at least during their first years of life and that pregnant women and elderly should have access to vitamin D supplementation [36]. The recommended doses for adults vary widely (from 200 IU to 2000 IU/day) [16], in line with disagreement regarding the minimal desirable serum concentration of 25(OH)D (Table 1). All guidelines agree that 25(OH)D levels <30 nmol/l (<12 ng/ml) should be avoided and that levels >75 nmol/l (>30 ng/ml) are sufficient. Adequate vitamin D status by this definition is probably reached by less than 50% of the world population, at least in winter [37]. In 2019, NHANES reported a prevalence of vitamin D deficiency (<30 nmol/l or 12 ng/ml) of 5% in the United States, with no change in prevalence between 2003 and 2014 [38].

**Table 1:** Definitions of vitamin D deficiency and adequacy according to different organizations (divide by 2.5 to convert from nmol/l to ng/ml).

Agency	Deficiency threshold (nmol/l)	Adequacy threshold (nmol/l)
European Food Safety Authority^a^	<30	>50
Nordic co-operation^b^	<30	>50
Institute of Medicine^c^	<30	>50
Endocrine Society [203]	<50	>75
European Calcified Tissue Society [204]	<50	

a Dietary reference values for vitamin D 4547 (European Food Safety Authority, 2016).

^b^Nordic nutrition recommendations 2012: integrating nutrition and physical activity (Nordic Council of Ministers, Copenhagen, 2012).

c Dietary reference intakes for calcium and vitamin D (Institute of Medicine, Washington, DC, 2010).

Vitamin D deficiency has been defined in the context of skeletal mineralization. Serum 25(OH)D level <30 nmol/l (<12 ng/ml) strongly increases the risk of rickets in children and osteomalacia in adults [39, 40]. This 25(OH)D level is also consistent with the threshold below which active intestinal calcium absorption decreases [41]. Vitamin D adequacy is most often defined as the vitamin D level that minimizes circulating PTH levels. Observational studies are inconsistent regarding this threshold, but clinical trials demonstrate that vitamin D supplementation results in a decrease in PTH if the baseline 25(OH)D is <50 nmol/l (<20 ng/ml) [42, 43]. Conversely, large-scale RCTs and Mendelian randomization studies failed to demonstrate a benefit of vitamin D supplementation for skeletal or extraskeletal health in adults with 25(OH)D levels >50 nmol/l (>20 ng/ml) [10]. Thus, in summary, the recommendation for the general population is to maintain serum 25(OH)D above 50–75 nmol/l, mainly to ensure skeletal health, as extraskeletal benefits of vitamin D supplementation remains controversial.

### 1.3 Vitamin D metabolism in CKD

#### Key evidence points

- CKD is a state of dysregulated vitamin D metabolism and vitamin D hyporesponsiveness.
- CKD is likely a state of extrarenal *CYP27B1* activation with extrarenal production of 1,25-dihydroxyvitamin D.

#### Background and rationale

Patients with CKD have a high prevalence of vitamin D deficiency [44–46]. Several underlying mechanisms have been proposed, including: (1) reduced ingestion of foods high in vitamin D content, (2) reduced endogenous synthesis of vitamin D_3_ in the skin due to reduced sunlight exposure or dermopathy, (3) increased FGF23-mediated vitamin D catabolism, and (4) increased losses, either through the kidneys (for instance, nephrotic syndrome [47, 48]) or the dialysate (peritoneal dialysis [49, 50]). It remains a matter of ongoing debate whether impaired liver 25-hydroxylase activity contributes to the low 25(OH)D levels in patients with CKD. Experimental kidney failure and hyperparathyroidism have been shown to decrease several *CYP450* isoforms involved in the 25-hydroxylation of vitamin D, but not *CYP2R1* [51]. Endotoxemia may be a common culprit of 25(OH)D deficiency in patients with CKD, diabetes, and non-alcoholic fatty liver disease [19, 52, 53]. Levels of 25(OH)D levels in the general population show seasonal fluctuations, vary across ethnic groups and according to dietary habits [54], and are lower in obese individuals [55]. There is no reason to assume that these sources of variability do not also apply in the setting of CKD. Studies comparing 25(OH)D levels in patients with CKD and otherwise healthy individuals, matched for key variables such as age, body mass index, ethnicity, and season, are scarce [44, 56]. As such, it is hard to estimate the direct impact of CKD on vitamin D deficiency.

Levels of 1,25(OH)_2_D decline as kidney failure progresses [44]. This decline seems mainly driven by the rising levels of FGF23, as FGF23-neutralizing antibodies have been shown to normalize serum 1,25-dihydroxyvitamin D levels in experimental models of CKD [57, 58]. Cohort studies also show decreasing levels of the catabolic product 24,25(OH)_2_D paralleling kidney function decline [59], suggesting overall impaired *CYP24A1* activity, possibly as a consequence of reduced 1,25(OH)_2_D levels.

The impact of CKD on extrarenal vitamin D metabolism is not well-defined, but it may be hypothesized that there is an increase in the extrarenal contribution to circulating levels of 1,25(OH)_2_D as kidney function declines. CKD enhances both the expression and efficiency of *CYP27B1* in peripheral blood monocytes as compared to healthy volunteers [23, 60]. Microinflammation, a common finding in patients with CKD [53], is a plausible driver of increased extrarenal *CYP27B1* activity [23]. Of interest, the extrarenal production of 1,25(OH)_2_D seems to be highly substrate-driven, which may have implications for target levels of 25(OH)D in CKD [29]. The cellular uptake of 25(OH)D and its affinity for *CYP27B1*, conversely, are impaired in CKD, and may be partly restored by 1,25(OH)_2_D supplementation [60]. In addition to a decreased production of 1,25(OH)_2_D, altered expression of the VDR or altered binding properties of the hormone receptor complex to DNA could also contribute to impaired 1,25(OH)_2_D signalling in CKD [61, 62]. Thus, CKD qualifies as a state of overall stagnant vitamin D metabolism, where both production and degradation of 1,25(OH)_2_D are suppressed [59]. The clinical systemic implications of extra-renal production of 1,25(OH)_2_D remains unclear.

### 1.4 Vitamin D metabolism in incident kidney transplant recipients

#### Key evidence points

- 25(OH)D levels show a transient decline early after kidney transplantation.
- Renal production of 1,25(OH)_2_D recovers after kidney transplantation paralleling kidney graft function.

#### Background and rationale

Low levels of vitamin D are common after kidney transplantation [63–65], despite an increased focus on supplementation in recent years [66]. Longitudinal studies show a transient decline in 25(OH)D levels shortly after transplantation [67, 68], which may be related to decreased vitamin D production in the skin (reduced sunshine exposure), impaired hepatic 25-hydroxylase activity (glucocorticosteroids [20]) or increased degradation (mediated by FGF23). Parallel to the recovery of kidney function, and progressive decreases in PTH and FGF23, 1,25(OH)_2_D levels rapidly increase from 3 months post-transplant onwards [29]. Of interest, patients who were able to maintain their 25(OH)D stores pre-transplant show a greater increase in 1,25(OH)_2_D levels in the early post-transplant period [67–69].

## Q2: What are the (pre)-analytical issues of vitamin D measurements and monitoring?

### 2.1 Analytical considerations

#### Key evidence points

- LC-MS/MS is the method of choice for measuring vitamin D metabolites, particularly in patients with CKD.
- The added value of the assessment of free vitamin D or metabolic ratios is uncertain.

#### Background and rationale

The most accurate approach to measure 25(OH)D in patients with advanced CKD is by liquid chromatography coupled with mass spectrometers in tandem (LC-MS/MS). This methodology brings an added advantage in its ability to quantify metabolites, for example the metabolite 24,25(OH)_2_D, which is produced by the enzyme *CYP24A1* and is the first step of the metabolization pathway of 25(OH)D. This metabolite is used to calculate the vitamin D metabolic ratio, which is further discussed below. Despite being the method of choice for quantifying 25(OH)D, LC-MS/MS is not widely implemented in clinical practice. Instead, many laboratories rely on commercially available immunoassays. Unfortunately, the vast majority of these immunoassays are inaccurate in the context of advanced CKD [70, 71]. A notable under-recovery, exceeding 20% of the metabolite, is observed, with no clear explanation beyond the alteration in the uremic matrix of these patients, which diverges from the matrix typically used for assay calibration, resulting in what is referred to as ‘matrix effects’. As a consequence, the traditional cut-offs used to delineate vitamin D sufficiency and insufficiency should probably not be applied to these immunoassays, even if they adhere to full standardization according to the Vitamin D Standardization Program criteria and are impeccably traceable to reference methods. Additionally, immunoassays do not uniformly quantify vitamin D_2_ metabolites (such as ergocalciferol), contributing to an elevated level of uncertainty in the reported values.

The ratio between the catabolic product 24,25(OH)_2_D and 25(OH)D, termed the *vitamin D metabolic ratio*, has been proposed as a measure of vitamin D status, with the rationale that lower levels of the catabolic product would indicate functional vitamin D deficiency [72, 73]. As discussed in the previous section, vitamin D catabolism is certainly affected by CKD, with levels of 24,25(OH)_2_D decreasing as kidney function declines [74, 75]. The 24,25(OH)_2_D response to vitamin D supplementation is also less pronounced in patients with CKD compared to kidney-healthy populations [76, 77]. In keeping with this, the vitamin D metabolic ratio decreases more prominently than 25(OH)D levels as CKD progresses [78]. However, whether this decrease is due to functional vitamin D deficiency or rather *CYP24A1* dysfunction in CKD is not clear. Reduced 25(OH)D clearance has been demonstrated in patients with CKD [79], indicating the latter and making the interpretation of metabolic ratios in CKD difficult. In one of the few studies to investigate the potential clinical value of the vitamin D metabolic ratio in CKD, a lower ratio was associated with cardiovascular events; however, the association disappeared after adjusting for kidney function [80].

The majority of vitamin D metabolites circulate bound to DBP and albumin (>99%) and, according to the free hormone hypothesis, may not be able to exert biological effects in most tissues. The utility of determining free vitamin D is a subject of ongoing debate [81]. The free fraction of vitamin D can be estimated mathematically through an equation that takes into account the levels of 25(OH)D, albumin, and DBP, along with affinity coefficients, akin to the approach published by Vermeulen *et al*. for free testosterone [82]. DBP should be measured using LC-MS/MS or a method utilizing polyclonal antibodies to avoid under-recoveries linked to DBP polymorphism. An LC-MS/MS method for DBP quantitation has been published [83] and international standards are readily available. In addition to the analytical considerations, the calculations utilized are based on multiple assumptions and have not been validated in different disease conditions [81]. Another option for direct measurement of free vitamin D is through a commercially available two-step competitive enzyme-linked immunosorbent assay [84]. In this approach, a monoclonal antibody against 25(OH)D is bound to a solid phase, which is supposed to selectively capture the free forms while excluding the bound ones. Subsequently, the quantification of this free form is achieved with a spectrophotometer. Alternatively, free vitamin D can be determined by an LC-MS/MS method, utilizing an equilibration dialysis step for sample preparation [85]. Of note, a recent comparison of both ‘direct’ methods revealed significant differences [85]. Measurement of free vitamin D is currently not recommended for the healthy background population [8], and with only a handful of studies investigating free vitamin D in CKD [86–88], there is insufficient evidence to consider the clinical utility in this setting.

Measuring 1,25(OH)_2_D is more complex than 25(OH)D as it circulates in the picomolar range, at concentrations approximately 1000 times lower than its precursor. Unlike the latter, there is currently no reference method available for 1,25(OH)_2_D. Given the lack of clinical support for determining 1,25(OH)_2_D in CKD, analytical challenges will not be further discussed.

In summary, 25(OH)D remains the preferred biomarker of vitamin D status. LC-MS/MS is the recommended method for determining vitamin D metabolites in CKD, particularly in later stages, but may not (yet) be widely available in clinical practice. Commercially available immunoassays should adhere to the current analytical standards and be traceable by the Vitamin D Standardization Program.

### 2.2 Monitoring strategy

#### Clinical practice points

- In patients with CKD G2-5D, we suggest measuring 25(OH)D concentrations at first presentation, with repeated analysis 3 months after any changes to vitamin D supplementation, and at least annually thereafter.
- Routine monitoring of 1,25(OH)_2_D levels, free vitamin D levels, and ratios between vitamin D metabolites are not recommended.

#### Background and rationale

Given the high prevalence of vitamin D deficiency and secondary hyperparathyroidism, and the apparent substrate dependence of *CYP27B1* activity in CKD, monitoring of vitamin D status seems reasonable in these patients. A tailored approach might be preferred, but practical guidance from clinical trials is lacking. Pragmatically, we suggest measuring 25(OH)D in patients with CKD G2-5D at first presentation and annually thereafter. A repeated analysis 3 months following 25(OH)D targeting intervention (for example, change of supplementation dose or interval) may be advised to assess treatment response, as both the supplement chosen (particularly with over the counter dietary supplements) and several patient-related factors (obesity, proteinuria, dialysis regime, baseline 25(OH)D level) can influence the response between dose given and increase in 25(OH)D levels. Potential benefits should be weighed against increased costs. These issues are further discussed in Section 5. We do not recommend the monitoring of 1,25(OH)_2_D, free vitamin D, or the ratio between 24,25(OH)_2_D and 25(OH)D in daily clinical practice pending additional evidence clarifying their clinical value.

## Q3: Is there evidence for benefit of vitamin D supplementation on bone and mineral metabolism in CKD?

### 3.1 Vitamin D supplementation and PTH control in CKD

#### Key evidence points

- Vitamin D supplementation improves control of secondary hyperparathyroidism in adults and children with CKD.

#### Clinical practice points

- We recommend supplementing vitamin D to >75 nmol/l (>30 ng/ml) in adults and children with CKD G2–5D to delay the onset, or improve the control, of secondary hyperparathyroidism, recognizing that the effect in CKD G5–5D is uncertain.
- We suggest replenishing vitamin D before using an active vitamin D compound in CKD G2–3 and before or concomitantly with an active vitamin D compound in CKD G4–5D.

#### Background and rationale

Vitamin D deficiency is a well-known cause of secondary hyperparathyroidism, and the inverse relationship between PTH and 25(OH)D levels is also apparent in CKD [89, 90]. A recent meta-analysis found a significant reduction in PTH by supplementation with vitamin D in CKD G3–4, but noted small study sizes and heterogeneity in the supplement chosen (cholecalciferol, ergocalciferol, or calcifediol), dosages used, and duration of the studies to be important limitations [91]. Not all studies were placebo-controlled, and some included participants regardless of vitamin D status at baseline [92–94]. Considering placebo-controlled trials in more detail, PTH reduction seems evident when achieving a target 25(OH)D >75 nmol/l (>30 ng/ml), particularly when using cholecalciferol (Table 2) [92, 95–111]. Ergocalciferol appears less efficient in achieving PTH reduction despite sufficient increases in 25(OH)D [93, 112–115]. In a head-to-head comparison, cholecalciferol resulted in greater increases in 25(OH)D levels in patients with CKD G3–5 compared to an equal dose of ergocalciferol, and only cholecalciferol resulted in PTH suppression [101]. Increased catabolism of vitamin D_3_ metabolites induced by ergocalciferol has been proposed as the explanation for the difference in clinical efficacy between these two vitamin D compounds [115]. In addition, three RCTs demonstrated PTH reduction with extended release calcifediol compared to placebo in patients with CKD G2–4 [116–119]. Very high levels of 25(OH)D were achieved in these trials, with successive PTH reduction, leading the authors to argue that a higher 25(OH)D target may be appropriate in CKD.

**Table 2:** Randomized trials of vitamin D supplementation on parathyroid hormone (PTH) levels in patients with chronic kidney disease (CKD; divide by 2.5 to convert from nmol/l to ng/ml).

Study	*N*	Population, CKD grade	Supplement and dose	Equivalent daily dose	Duration	Baseline 25(OH)D (nmol/l)	Follow-up 25(OH)D (nmol/l)	PTH change
Dogan 2008 [99]	70	Adults, G3–4	Chol 300 000 IU/month versus none	10 000	12 weeks	21/17	45/18	↓
Chandra 2008 [100]	34	Adults, G3–4	Chol 50 000 IU/week versus placebo	7143	12 weeks	43/47	123/49	─
Alvarez 2012 [92]	46	Adults, G2–3	Chol 50 000 IU/week for 12 weeks > 50 000 IU/2 weeks for 40 weeks versus placebo	7143 then 3572	52 weeks	67/80	107/66	↓
Marckmann 2012 [95]	27	Adults, G1–5D	Chol 40 000 IU/week versus placebo	5715	8 weeks	39/29	166/22	↓
Petchey 2013 [94]	28	Adults, G3–4	Chol 2000 IU/day versus placebo	2000	24 weeks	95/88	146/81	─
Kumar 2017 [98] and Yadav 2018 [97]	120	Adults, G3–4	Chol 300 000 IU every 8 weeks versus placebo	5357	16 weeks	33/33	95/37	↓
Westerberg 2018 [96]	95	Adults, G3–4	Chol 8000 IU/day versus placebo	8000	12 weeks	58/57	162/57	↓
Shroff 2012 [120]	47	Children, G2–4	Ergo 2–8000 IU/day for 3 months, then 2000 IU/day for 9 months versus placebo	2–8000 then 2000	52 weeks	50/52	83/	↓
Gravesen 2013 [93]	43	Adults, G4–5	Ergo 50 000 IU/week versus none	7143	8 weeks	63/59	129/60	─
Dreyer 2014 [112]	38	Adults, G3–4	Ergo 50 000 IU/week for 1 month then 50 000 IU/month versus plaebo	7143, then 1667	24 weeks	30/25	80/25	─
Sprague 2014 [117]	78	Adults, G2–4	Calcifediol (ERC) 30, 60 or 90 μg versus placebo	─	6 weeks	55/50	93–212/46	↓
Sprague 2016 [116]216	213	Adults, G3–4	Calcifediol (ERC) 30 or 60 μg versus placebo	─	26 weeks + 26 weeks open-label extension	50/50	125–172/50	↓

Abbreviations: ERC = extended release calcifediol; HD = hemodialysis; PD = peritoneal dialysis; PTH = parathyroid hormone.

Most, but not all, studies specifically included patients with vitamin D insufficiency, typically defined as 25(OH)D < 50–75 nmol/l (20–30 ng/ml), and some further specified that patients should have secondary hyperparathyroidism [96, 100]. One study reported no overall difference in PTH levels after cholecalciferol supplementation in CKD G2–3, except in the subgroup of patients with hyperparathyroidism at baseline [92]. This raises the question of whether vitamin D monitoring and supplementation should be reserved for patients with secondary hyperparathyroidism. However, in children with CKD, the time to manifest hyperparathyroidism could be delayed with vitamin D supplementation [120], and in adults, vitamin D supplementation was found to be equally effective to an active vitamin D compound in achieving PTH target levels in CKD G3–4 [114], which provides arguments for a more pre-emptive approach.

The effect of vitamin D supplementation on PTH control in CKD G5–5D is less certain than in earlier stages, with discordant findings in available trials (Table 3). Changes in concomitant medical therapy for secondary hyperparathyroidism (active vitamin D compounds, calcimimetics) may, to some degree, obscure the effect of vitamin D supplementation on PTH. Indeed, some studies reported decreased use of active vitamin D compounds in the intervention arm [103, 105]; however, others reported no change in dose [109, 110, 113]. One study demonstrated that 1,25(OH)_2_D levels increased to a similar degree with cholecalciferol versus alfacalcidol [102], and another that PTH reduction was greater with the combination of paricalcitol and cholecalciferol, compared to the active vitamin D compound alone [106], which argues for a role of ensuring vitamin D sufficiency even in kidney failure. PTH reduction was also reported with vitamin D supplementation in prospective studies that did not include a control group [121–123]. Thus, the effect of vitamin D supplementation on PTH control is clearly less certain in CKD G5–5D than in earlier stages. Considering the substrate-dependence of CYP27B1 activity in CKD G5–5D [29, 124], a higher 25(OH)D treatment target could be argued for; however, there is no solid evidence to support this. Given the tolerability of vitamin D supplementation and the potential benefit, it seems prudent to avoid vitamin D deficiency in CKD G5–5D.

**Table 3:** Randomized trials of vitamin D supplementation on parathyroid hormone (PTH) levels in adults with kidney failure (divide by 2.5 to convert from nmol/l to ng/ml).

Study	*N*	CKD grade	Supplement and dose	Equivalent daily dose (IU/day)	Duration	Baseline 25(OH)D (nmol/l)	Follow-up 25(OH)D (nmol/L)	PTH change
Marckmann 2012 [95]	25	G1–5D (HD)	Chol 40 000 IU/week versus placebo	5715	8 weeks	21/36	136/26	─/↓^a^
Delanaye 2013 [111]	43	G5D (HD)	Chol 25 000 IU/2weeks versus placebo	1786	52 weeks	30/30	80/44	↓
Hewitt 2013 [110]	60	G5D (HD)	Chol 50 000 IU/week for 8 weeks then /month versus placebo	7143 then 1667	24 weeks	43/43	88/40	─
Li 2014 [109]	96	G5D (HD)	Chol 50 000 IU/week for 6 weeks then 10 000 IU/week versus none	7143 then 1429	52 weeks	34/33	106/40	─
Massart 2014 [108]	55	G5D (HD)	Chol 25 000 IU/week versus placebo	3571	24 weeks	46/43	88/41	─
Bhan 2015 [113]	105	G5D (HD)	Ergo 50 000 IU/week or /month versus placebo	7143 or 1667	16 weeks	55/56/54	125 or 96/69	─
Zheng 2016 [106]	60	G5D (HD)	Chol 5000 IU/week versus none	714	16 weeks	49/49	76/49	↓
Wang 2016 [107]	746	G5D	Chol 50 000 IU/week versus placebo	7143	52 weeks	55/58	103/58	↓
Zheng 2018 [105]	60	G5D (HD)	Chol 5000 IU/day versus none	5000	24 weeks	46/48	94/58	↓
Alshahawey 2021 [104]	60	G5D (HD)	Chol 200 000 IU/month versus placebo	6667	12 weeks	45/47	80/47	↓
Matuszkiewicz-Rowinska 2022 [102]	62	G5D (HD)	Chol 4000 IU ×3/week versus alfacalcidol or placebo	1714	12 weeks	32/38	78/33	─
Brimble 2022 [103]	65	G5D (PD)	Chol 50 000 IU/week for 8 weeks then 10 000 IU/week versus placebo	7143 then 1429	52 weeks	52/49	69/40	─

Abbreviations: Chol = Cholecalciferol; Ergo = rrgocalciferol; HD = hemodialysis; PD = peritoneal dialysis; PTH = parathyroid hormone.

^a^Prevalence of hyperparathyroidism decreased with cholecalciferol.

All studies included adult patients; no trials with children in CKD G5D were identified.

The ESPN has published comprehensive clinical practice recommendations for both vitamin D supplementation and active vitamin D therapy in children with CKD [5, 125]. In children with CKD G2–4, ergocalciferol given to a target of 25(OH)D >75 nmol/l (>30 ng/ml) delayed the onset of secondary hyperparathyroidism [120]. A second study, including children with CKD G2–4, also demonstrated PTH reduction with high-dose cholecalciferol with approximately half of the cohort achieving a 30% reduction in PTH levels [126]. Lastly, in the C3 trial [112, 113], which included children with CKD G2–4, lower PTH levels were seen when achieving 25(OH)D >75 nmol/l (>30 ng/ml) [127], and there was an inverse relationship between the 25(OH)D increase and the PTH decrease [128].

In conclusion, for both children and adults with CKD, vitamin D supplementation to a 25(OH)D target >75 nmol/l delays the onset, or improves the control, of secondary hyperparathyroidism. Although the effect is less certain in CKD G5–5D, there is currently insufficient evidence to suggest differential 25(OH)D targets in patients with kidney failure.

### 3.2 Vitamin D supplementation and bone outcomes in CKD

#### Key evidence points

- There is no evidence for a benefit of vitamin D supplementation alone on the risk of bone loss and fractures in adults or children with CKD, or for improved growth in children with CKD.

#### Background and rationale

Vitamin D supplementation may benefit skeletal health indirectly through improved intestinal calcium absorption and suppression of secondary hyperparathyroidism [129], or directly through effects on bone mineralization [130]. However, whether fracture risk is reduced by vitamin D supplementation at the population level remains controversial [10]. A recent meta-analysis attempted to pool fracture data from trials of vitamin D supplementation in CKD, but the low number of events in each trial renders these analyses inconclusive [131]. Of two studies identified investigating the effect of vitamin D supplementation on intermediate bone outcomes (bone imaging), one was a combined intervention with calcium and cholecalciferol given to frail, elderly women with CKD G1–3 [132]. This study concluded that there was an overall positive treatment effect on distal radius BMD, which did not differ based on baseline kidney function [132]. A second study included patients with CKD G5D receiving hemodialysis and investigated an intervention of cholecalciferol 5000 IU/day versus no vitamin D supplement on top of cinacalcet and calcitriol, and reported a non-significant trend towards improved BMD at the femoral neck with cholecalciferol [105].

Vitamin D deficiency has been associated with bone mineralization defects both in the general population [133] and in CKD [134], but to our knowledge, there are no trials that inform on the effects of vitamin D supplementation on bone histomorphometry in (contemporary) patients with CKD. Trials of active vitamin D compounds demonstrate improvements in bone histology both in early [135] and late [136] stages of CKD, but it is difficult to separate any independent and direct effects of vitamin D in bone from those mediated by improved control of hyperparathyroidism [135]. To our knowledge, there are no trials to inform on the effect of vitamin D supplementation on bone outcomes, including growth, in children with CKD. Thus, although there is biological plausibility, there is insufficient evidence to support beneficial effects on skeletal health by vitamin D supplementation beyond what results from improved PTH control.

### 3.3 Vitamin D metabolism, PTH control, and bone outcomes in kidney transplant recipients

#### Key evidence points

- Vitamin D supplementation improves the control of hyperparathyroidism after kidney transplantation.
- Vitamin D supplementation may reduce the risk of bone loss and fractures after kidney transplantation.

#### Clinical practice points

- We suggest supplementing vitamin D to >75 nmol/l (>30 ng/ml) in kidney transplant recipients to reduce the risk of bone loss and fractures.

#### Background and rationale

As in the general population, low levels of vitamin D associate with poor outcomes post-transplant [65] including osteoporosis [137] and disturbed bone turnover and mineralization [138]. Five trials investigated the effect of vitamin D supplementation on bone or related biochemical outcomes after kidney transplantation (Table 4) [139–143], two of which were RCTs [140, 144]. Tsujita *et al*. randomized recipients of a living donor kidney transplantation from 1 month post-transplant to either cholecalciferol 4000 IU/day or placebo for 12 months [140]. Baseline 25(OH)D was notably low, at 25 nmol/l (10 ng/ml). Supplementation resulted in a substantial increase in 25(OH)D levels (to 100 nmol/l or 40 ng/ml) and a greater decrease in PTH than in the placebo group. The treatment effect on PTH was more pronounced in patients with good kidney graft function and lower baseline levels of calcium or 25(OH)D. Lumbar spine BMD was better preserved with vitamin D supplementation, which could be partly explained by the lower PTH levels. In the VITamin D supplementation in renAL transplant recipients (VITALE) study, prevalent kidney transplant recipients with stable kidney graft function and 25(OH)D levels <75 nmol/l (<30 ng/ml) were randomized to either high or low dose of cholecalciferol for 24 months [139]. The primary outcome was a composite of diabetes mellitus, cardiovascular disease, cancer, or death (discussed in the next section), and symptomatic fractures were included as a secondary outcome. Fracture incidence was significantly lower in the high-dose cholecalciferol group, although the number of events were small (3 versus 12 events). There was a marginal PTH lowering effect of high-dose cholecalciferol, but no effect on BMD in a subgroup of patients with available dual-energy X-ray absorptiometry scans. Two additional studies investigating effects of calcium and vitamin D supplementation on BMD in the first post-transplant year reported diverging results [142, 143].

**Table 4:** Randomized controlled trials of vitamin D supplementation after kidney transplantation with bone or biochemical outcomes (divide by 2.5 to convert from nmol/l to ng/ml).

Study	*N*	Population	Intervention	Duration	Equivalent daily dose (IU/day)	Baseline 25(OH)D (nmol/l)	Follow-up 25(OH)D (nmol/l)	PTH	BMD	Fracture
Courbebaisse 2023 [144]	536	Prevalent, 12–48 months post-TX	Chol 100 000 IU (*high*) versus 12 000 IU (*low*) every 2 weeks for 2 months, then /month	24 months	7143 then 3333 versus 857 then 400	51/48	108/63	↓	No effect (LS, FN)	Reduced risk (clinical fractures)
Tsjuita 2022 [140]	193	Incident, 1 month post-TX	Chol 4000 IU/day versus placebo	12 months	4000	25/25	100/35	↓	Improved (LS)	Not reported
Courbebaisse 2009 [141]	94	Incident, 3 months post-TX	Chol 100 000 IU/2 weeks for 2 months followed by every 2 months versus none	12 months	7143 then 1667	35/30	75/32	↓	Not reported	Not reported
Wissing 2005 [142]	90	Incident, from TX	Chol 25 000 IU/month with 400 mg/day Ca versus Ca alone	12 months	822	61/49	67/41	↓	No effect (LS, FN)	Not reported
Talalaj 1996 [143]	71	Incident, from TX	Calcidiol 40 μg/day + Ca 3 g/day versus none	12 months	1600	32/41	72/48	_	Improved (LS, FN)	Reduced risk (incident VFx)

Data are mean or median, as given in the source data.

Abbreviations: BMD = bone mineral density; Calc = calcidiol; Chol = cholecalciferol; FN = femoral neck; LS = lumbar spine; N/A = not available; VFx = vertebral fracture; TX = transplantation.

In conclusion, although the evidence is sparse, two well-performed RCTs indicate benefit of vitamin D supplementation on bone outcomes after kidney transplantation, likely mediated through improved control of hyperparathyroidism.

## Q4: Is there evidence for benefit of vitamin D supplementation on cardiovascular outcomes and all-cause mortality in CKD?

### 4.1 Vitamin D supplementation and cardiovascular outcomes and mortality in CKD

#### Key evidence points

- There is no evidence for a benefit of vitamin D supplementation on cardiovascular outcomes or all-cause mortality in CKD.
- No studies revealed clinically relevant cardiovascular safety concerns of vitamin D supplementation.

#### Background and rationale

**Figure 4:**
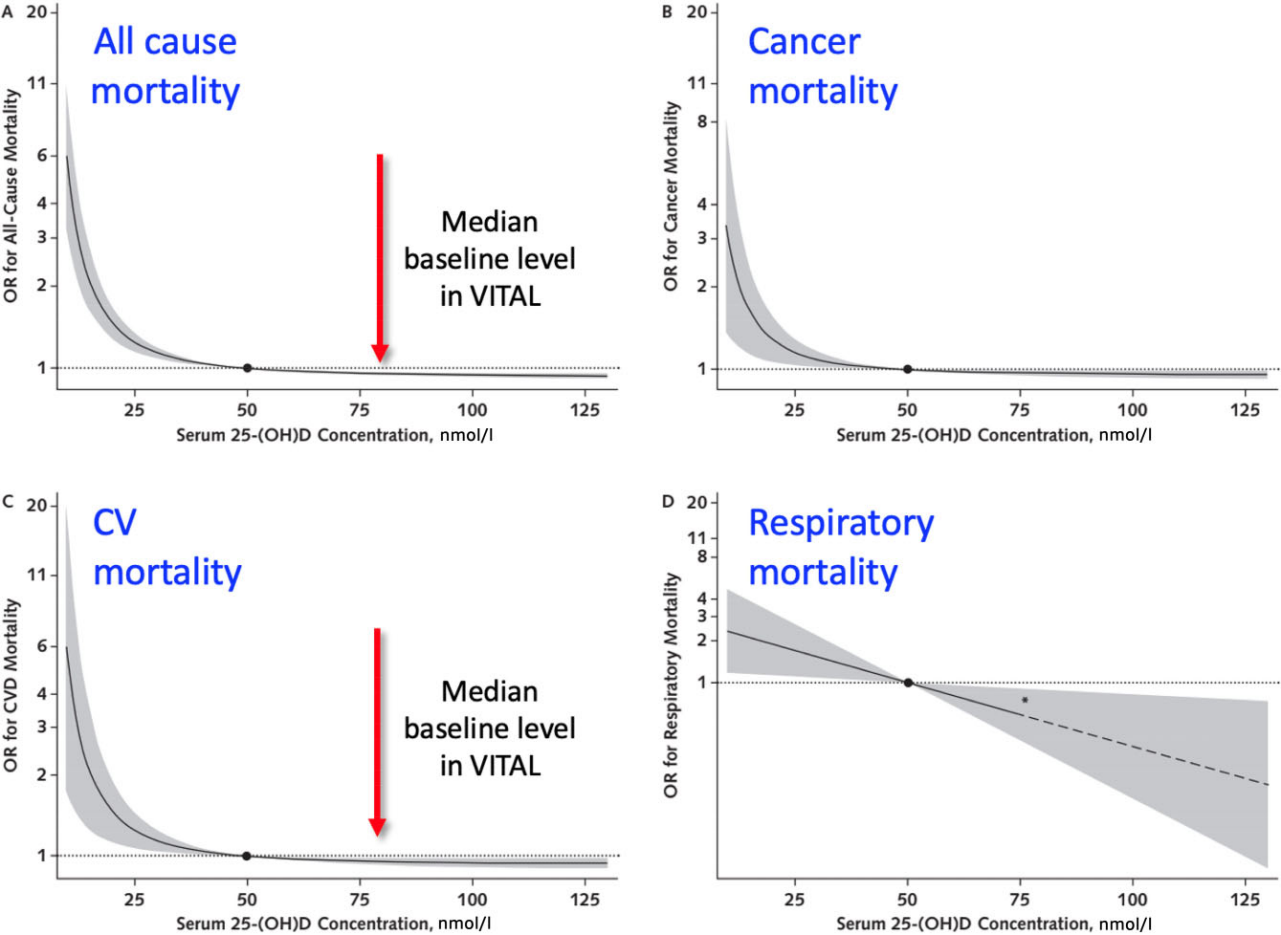
Association between predicted levels of 25(OH)D, based on genetic determinants, and all-cause and cause-specific mortality. Figure from a Mendelian randomizataino study utilizing the UK Biobank [155]. Arrows indicate the median baseline level in the VITamin D and OmegA-3 TriaL (VITAL) [151]. CV = Cardiovascular (divide by 2.5 to convert from nmol/l to ng/ml).

It has been hypothesized that vitamin D deficiency contributes to a range of illnesses, beyond and unrelated to its role in disturbed mineral homeostasis and bone disease. These pleiotropic effects are assumed to also involve the cardiovascular system. This hypothesis is based on the finding that the VDR is expressed on many cells, including in cardiovascular tissues [145], and on observational data that demonstrate an inverse association between 25(OH)D levels and the incidence of cardiovascular events and mortality, both in the general population [146, 147] and in patients with CKD [148, 149]. However, systematic reviews and meta-analyses on the effects of vitamin D supplementation on these outcomes in CKD have provided contradictory results [131, 150]. Importantly, recent large-scale intervention studies in the general population essentially refute the causality of this association. The VITAL trial recruited over 25 000 participants who were randomized to either 2000 IU cholecalciferol or placebo (and to omega 3 fatty acids or placebo in a 2 × 2 factorial design) [151]. The study was negative on its primary (incident invasive cancer or MACE) and secondary endpoints, and there was no effect modifications of any subgroup on MACE. In addition, there was no signal of beneficial effect of vitamin D supplementation within the subgroup with baseline 25(OH)D levels <50 nmol/l (<20 ng/ml), although the number of events were too small to reliably show a difference between the study arms. The absence of effect on cardiovascular morbidity and mortality rates confirmed findings from previous large trials in the general population [152, 153]. Moreover, a subsequent study from Australia, the D-Health Trial, of comparable size to VITAL, found no indication of any benefit of vitamin D supplements on mortality risk, including cardiovascular mortality in the older general population [154]. One conclusion from these studies may be that vitamin D deficiency has no causal role in these non-skeletal outcomes. However, a recent Mendelian randomization study from the UK Biobank provided some evidence to the contrary [155]. Studying the association between *predicted* levels of 25(OH)D, based on a wide range of genetic determinants, and all-cause and cause-specific mortality, a sharp increase in risk emerged with 25(OH)D levels <25 nmol/l (<10 ng/ml), that is, severe vitamin D deficiency (Fig. 4). This ‘inflection level’ corresponds to the level of 25(OH)D below which 1,25(OH)_2_D production becomes substrate dependent [156]. Importantly, baseline 25(OH)D levels of patients enrolled in the VITAL study were well above the range where according to the UK Biobank study, 25(OH)D levels really matter (Fig. 4). Thus, the population included in the VITAL study may have been too vitamin D replete to benefit from the intervention.

Next, the question arises whether data obtained in the general population can be applied to the CKD setting. The VITAL investigators failed to demonstrate effect modification by estimated glomerular filtration rate (eGFR) [157], but the number of participants with an eGFR < 60 was low, limiting statistical power. So far, only one prospective trial examined the potential benefits of vitamin D supplementation on cardiovascular outcomes in a CKD population. The VITALE study randomized 538 kidney transplantation recipients to low- or high-dose cholecalciferol for two years [139]. There was no difference between groups in the time to occurrence of the composite endpoint of diabetes, cancer, MACE, or death, or between any of the individual components [139]. This study also warrants cautious interpretation given the relatively small sample size and short follow-up time.

One may speculate that the abovementioned ‘inflection level’ is either non-existing or higher in the setting of CKD, acknowledging the marked impact of CKD on vitamin D metabolism with 1,25(OH)_2_D becoming increasingly substrate dependent as kidney function declines. This reasoning fuels the hope that the currently recruiting Survival Improvement with Cholecalciferol in Patients on Dialysis (SIMPLIFIED) study will yield meaningful results. SIMPLIFIED is a prospective, randomized, open-label, multicenter trial of cholecalciferol versus standard care in patients receiving dialysis. Approximately 4200 subjects from 65 dialysis units in the United Kingdom will be included in the trial, and the primary objective is to determine the effect of cholecalciferol 60 000 IU every 2 weeks on patient survival [158].

Besides the above-mentioned trials on patient-relevant endpoints, a range of studies assessed the effects of vitamin D supplements on surrogate markers of cardiovascular health in people with CKD (Table 5), including pulse wave velocity (PWV; reflecting arterial stiffness) [95, 110, 159, 160] progression of arterial calcification [161], flow-mediated vasodilation (reflecting endothelial function) [98], left ventricle mass index [162], and blood pressure [92, 95, 163, 164]. Overall, these studies found no benefit of vitamin D supplementation, with just a few nuances. In the study by Levin *et al*. the group allocated to calcifediol had an improved PWV after 6 months as compared to placebo [159]. However, there were substantial differences of PWV at baseline, and when results were adjusted for that, the benefit was lost. The other exception was the study by Kumar *et al*. studying the effects of high dose cholecalciferol (300 000 IU administered twice) on flow-mediated vasodilation in patients with CKD [98]. This study found a 5.5% improved endothelial function as compared to placebo after 16 weeks.

**Table 5:** Randomized trials investigating the effect of nutritional vitamin D supplementation on intermediate cardiovascular endpoints in patients with chronic kidney disease (CKD; divide by 2.5 to convert from nmol/l to ng/ml).

Study	*N*	CKD grade	Supplement and dose	Equivalent daily dose (IU/day)	Duration	Baseline 25(OH)D (nmol/l)	Follow-up 25(OH)D (nmol/l)	Effect on cardiovascular outcome
Alvarez 2012 [92]	46	G2–3	Chol 50 000 IU/week for 12 weeks, then/2 weeks versus placebo	7143 then 3572	52 weeks	67/67	100/80	Blood pressure: ─
Courbebaisse 2023 [139]	536	G1–5T	Chol 100 000 IU versus 12 000 IU both/2 weeks for 2 month, then /month	7143 then 3333 *versus* 857 then 400	104 weeks	50/50	107/62	MACE: ─
Levin 2017 [159]	87	G3b–4	Calcifediol 5000 3×/week versus placebo	2143	26 weeks	67/67	235/65	PWV: ─/↓
Banerjee 2021 [162]	48	G3–4	Chol 100 000 IU at week 0, 4, 8, 12, 24, 42 versus placebo	1648	52 weeks	46/37	∼75/∼40	LVMI: ─
Das 2023 [160]	100	G3	Chol 3000 IU/week for 8 weeks versus placebo	429	24 weeks	42/42	90/60	PWV: ─
Dreyer 2014 [112]	38	G3–4	Ergo 50 000/week for 1 month, then/month versus placebo	7143, then 1667	26 weeks	30/25	80/25	Endothelial function: ↑
Hewitt 2013 [110]	66	G5D(HD)	Chol 50 000/week for 8 weeks, then /month versus placebo	7143 then 1667	26 weeks	45/40	88/40	PWV: ─
Kumar 2017 [98] & Kaur 2022 [205]	120	G3–4	Chol 300 000 week 0 and 8 versus placebo	5357	16 weeks	32/32	94/34	FMD: ↑; PWV:↓
Liyanage 2017 [163] & 2018 [206]	85	G1–3	Chol 50 000 im/month versus placebo	1667	52 weeks	55/50	82/46	Blood pressure: ─
Marckmann 2012 [95]	52	G1–5D	Chol 40 000/week versus placebo	5715	8 weeks	24/33	155/24	Blood pressure, PWV: ─
Massart 2014 [108]	55	G5D(HD)	Chol 25 000/week versus placebo for 13 weeks, then 26 weeks to target	3571 (individualized)	39 weeks	42/45	65/65	Aortic calcification: ─
Samaan 2018 [161]	47	G3–4	Chol 50 000/month versus placebo	1667	78 weeks	50/55	95/75	Coronary artery calcification score: ─

Abbreviations: Chol = Cholecalciferol, Ergo = ergocalciferol, HD = hemodialysis, MACE = major cardiovascular events, PWV = pulse wave velocity, FMD = flow-mediated vasodilatation, LVMI = left ventricular mass index.

All studies included adult patients with CKD; no studies with children were identified.

Of note, none of these studies revealed any safety issues with vitamin D supplements in terms of incidences of hypercalcemia or hyperphosphatemia. A few studies reported on the effects of vitamin D supplements on FGF23 (Supplementary Table S1) [92, 96, 98, 102, 104, 116, 117, 127, 128]. Overall, no effect was seen on FGF23 levels, although one study reported a transient increase in FGF23 in CKD patients with baseline vitamin D sufficiency (25(OH)D levels > 75 nmol/l) allocated to cholecalciferol [67]. A recent meta-analysis of randomized clinical trials did indicate an increase in FGF23 levels with vitamin D supplements in a wide range of study populations, most of them without CKD [165]. The effect was modest, with a weighted mean difference in FGF23 levels of +21 pg/ml; however, it was more pronounced in people with kidney or heart failure (+300 pg/ml). FGF23 has consistently been associated with dismal clinical outcomes, but it is still unclear if FGF23 should be a treatment target in CKD.

## Q5: What is the approach to the management and prevention of vitamin D deficiency in patients with CKD?

### 5.1 Pharmaceutical considerations

#### Clinical practice points

- We suggest using oral cholecalciferol to increase and maintain serum 25(OH)D concentration in the target range. If cholecalciferol is unavailable, oral ergocalciferol may be used in an equivalent dose.
- Oral administration of cholecalciferol should be preferred, with intramuscular administration reserved for patients with gastrointestinal malabsorption disorders.
- Vitamin D compounds should be administered by mouth and not through feeding tubes.

#### Background and rationale

Clinical evidence shows that nutritional vitamin D compounds are effective at increasing serum 25(OH)D levels in patients with CKD. Cholecalciferol seems to be more efficient than ergocalciferol, both for increasing serum 25(OH)D [166] and reducing serum PTH levels [101] (Table 6). This is consistent with findings in the general population (Supplementary Table S3) [167–169], and likely due to increased catabolism of vitamin D_3_ metabolites induced by ergocalciferol, as previously discussed.

**Table 6:** Randomiaed trials comparing different types of nutritional vitamin D in adults with chronic kidney disease (divide by 2.5 to convert from nmol/l to ng/ml).

Study	N	CKD stage	Baseline 25(OH)D (nmol/l)	Ergocalciferol dose	Cholecalciferol dose	Outcome
Wetmore 2016 [101]	44	G3–5	Ergo: 51 Chol: 52	50 000 IU/week for 12 weeks	50 000 IU/week for 12 weeks	Cholecalciferol yielded a greater mean change in 25(OH)D (113 ± 41 nmol/l) than ergocalciferol (77 ± 38 nmol/l) and was more efficient in decreasing PTH
Daroux 2013 [166]	39	G5D (HD)	50	200 000 IU/month given as 3× week, or /mo for 3 months	200 000 IU/month for 3 months	Cholecalciferol yielded a greater mean change in 25(OH)D (100 ± 33 nmol/l) than ergocalciferol (63 ± 23 nmol/l)

Abbreviations: 25(OH)D = 25-hydroxyvitamin-D; Chol = cholecalciferol: Ergo = ergocalciferol; HD = hemodialysis; IU = international unit; PTH = parathyroid hormone.

Studies of nutritional vitamin D have largely focused on the oral route of administration, with only one study in CKD providing comparative data for the intramuscular route (Supplementary Table S3) [170]. The pharmacokinetic profiles of vitamin D differ substantively between oral and intramuscular routes with respect to the rate at which peak levels are attained and decline, and therefore differences in when 25(OH)D levels were measured can confound comparisons. Thus, such comparisons need to be interpreted cautiously [170–172]. Despite this limitation, the data collectively show that both routes of administration lead to an increase in serum 25(OH)D levels [170–172]. However, intramuscular injections are invasive, and may cause pain, swelling, bleeding, and bruising at the injection site, and should therefore be reserved for patients with gastrointestinal malabsorption disorders. Vitamin D compounds are lipophilic, hence must be given by mouth and not through feeding tubes as they may adhere to the plastic [173], resulting in a lower and variable dose ingested.

Oral nutritional vitamin D products are commercially available in both pharmaceutical-grade and dietary supplements sold as over-the-counter preparations [174, 175]. Although pharmaceutical-grade vitamin D products are subject to the same rigorous regulatory manufacturing standards as other medicines, a number of studies have reported substantial variation in the actual vitamin D content compared to the value declared on the label (ranging from <10% to >200%) for over-the-counter vitamin D dietary supplements (Supplementary Table S4) [174–179]. Thus, monitoring the response on 25(OH)D levels within a reasonable time frame (arbitrarily set at 3 months, see Section 2.2) after initiation of therapy is advisable.

### 5.2 Recommended dosage regimens in CKD

#### Clinical practice points

- We suggest using oral cholecalciferol with once-daily, weekly, fortnightly, or monthly dosing schedules, adjusting the dose for baseline 25(OH)D levels and body size.
- In adults with CKD G2–5D or after kidney transplantation with serum 25(OH)D concentration <75 nmol/l (<30 ng/ml), we suggest an equivalent daily dose of 5000–7000 IU/day of cholecalciferol for a duration of 12 weeks to achieve the optimal target range. To maintain serum 25(OH)D concentration in the target range, we suggest continuous dosing with an equivalent daily dose of 2000 IU/day.
- In children with CKD G2–5D or after kidney transplantation with serum 25(OH)D concentration <75 nmol/l (<30 ng/ml), we suggest an equivalent daily dose of 3000–7000 IU/day of cholecalciferol for a duration of 12 weeks to achieve the optimal target range. To maintain serum 25(OH)D concentration in the target range, we suggest continuous dosing with an equivalent daily dose of 1000–2000 IU/day, adjusting for body size.
- We suggest avoiding exceeding doses of 100 000 IU given as a single dose.

#### Background and rationale

The dose of vitamin D is a key determinant of the rate at which serum 25(OH)D concentration increases, although wide variability is observed between dose given and 25(OH)D levels achieved in both patients with CKD [120] and in the general population [180]. Due to the heterogeneity in dosing regimens across studies (dose, frequency of dosing, duration of therapy) and variable patient-related factors (body weight, obesity, proteinuria, baseline 25(OH)D levels) that could affect the dose–response relationship [55, 127, 168, 181], determining the optimal dose is challenging [125, 131]. Certain patient groups such as those that are obese, have high-grade proteinuria, or receive peritoneal dialysis therapy may require higher doses of cholecalciferol to achieve target 25(OH)D levels. The association between obesity and lower vitamin D levels is well described for the background population [55, 182] and is also apparent in CKD [121]. Proposed mechanisms include effects of metabolic status on hydroxylation of vitamin D in the liver (discussed in Section Q1) and sequestration of vitamin D metabolites by adipose tissue. For glomerular diseases [127, 183] and peritoneal dialysis therapy [49, 50, 184], increased loss of DBP (in urine or dialysate) is hypothesized to be the reason for lower 25(OH)D levels and need for higher supplement doses to reach treatment targets.

**Figure 5:**
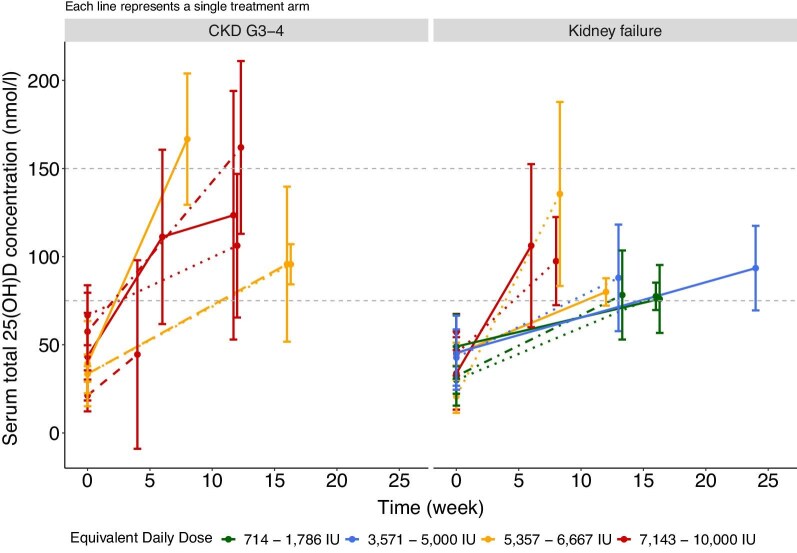
25(OH)D response of 16 study arms from randomized controlled trials of nutritional vitamin D in adults with CKD where the outcome was change in PTH levels (divide by 2.5 to convert from nmol/l to ng/ml). Studies included: Alvarez *et al*., 2012 [92]; Marckmann *et al*., 2012 [95]; Westerberg *et al*., 2018 [96]; Yadav *et al*., 2018 [97]; Kumar *et al*., 2017 [98]; Dogan *et al*., 2008 [99]; Chandra *et al*., 2008 [100]; Matuszkiewicz-Rowinska *et al*., 2021 [102]; Alshahawey *et al*., 2021 [104]; Zheng *et al*., 2018 [105]; Zheng *et al*., 2016 [106]; Massart *et al*., 2014 [108]; Li *et al*., 2014 [109]; Hewitt *et al*., 2013 [110]; Delanaye *et al*., 2013 [111].

**Figure 6:**
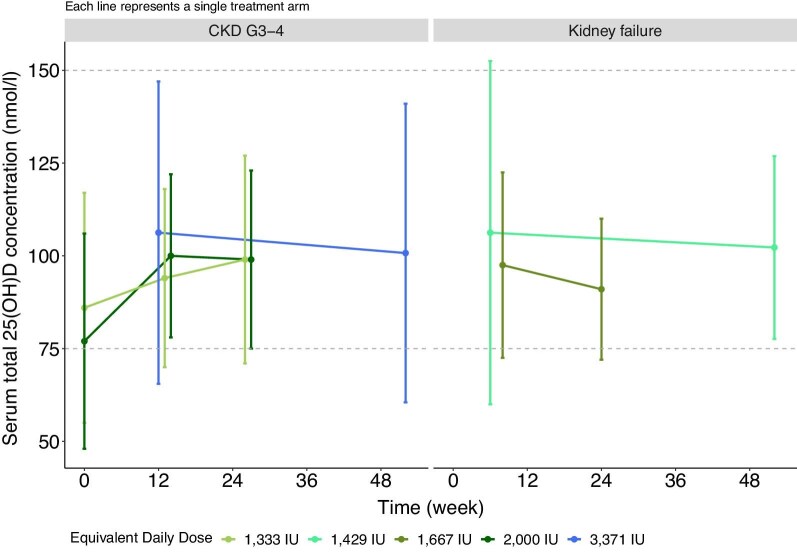
25(OH)D response of five study arms from randomized controlled trials of nutritional vitamin D in adults with CKD where doses to maintain 25(OH)D levels were investigated (divide by 2.5 to convert from nmol/l to ng/ml). Data for the initial period where higher doses of vitamin D were used in some of these studies are excluded for illustration purposes only. Studies included: Alvarez *et al*., 2012 [92]; Li *et al*., 2014 [109]; Hewitt *et al*., 2013 [110]; Mager *et al*., 2016 [208]. (Note: Mager *et al*., 2016 included patients with CKD G1–2.)

There is no clear consensus on optimal dosages of vitamin D supplementation for adults or children with CKD. A commonly employed strategy is to use higher dose for a variable duration of 4–12 weeks to increase serum total 25(OH)D concentration to the target range followed by a lower dose as maintenance therapy [125, 185]. The vitamin D doses used in RCTs in adults with CKD with PTH outcomes are shown in Tables 2 and 3 and Figs 5 and 6. There is no rationale for rapid correction of 25(OH)D deficiency, and the use of mega-doses is not recommended (see below). There is significant variation in the doses used in trials (equivalent daily dose: ∼700– 8000 IU), but collectively, it seems reasonable to suggest an equivalent daily dose of 5000–7000 IU/day of cholecalciferol for 12 weeks to achieve 25(OH)D > 75 nmol/l (>30 ng/ml). Of note, the average baseline 25(OH)D level in studies ranged from 33 to 68 nmol/l (13.2–27.2 ng/ml), and individuals with lower 25(OH)D may require longer duration and higher doses to achieve the same target range. Equally, variation in individual responses, as indicated by the large standard deviations, suggests that personalized dosing regimens may be needed. As discussed above, in patients with CKD, 25(OH)D *production* is not substrate dependent, and there is a non-linear correlation between the dose given and the 25(OH)D level achieved [120]. To maintain serum 25(OH)D > 75 nmol/l (>30 ng/ml), continuation with a lower dosing regimen is advocated. In the only study in adults with non-dialysis-dependent CKD that examined cessation of treatment, serum 25(OH)D levels declined substantially at 6 weeks following treatment cessation [101]. There are few studies examining the optimal dose needed to maintain serum 25(OH)D, but the available data suggest an equivalent daily dose of 2000 IU/day of cholecalciferol [92, 103, 109, 110].

In a randomized placebo-controlled trial of nutritional vitamin D in children with CKD, a modified version of the K/DOQI dosage recommendations was used, adjusting for both baseline 25(OH)D concentrations and the child's age. A target 25(OH)D > 75 nmol/l (>30 ng/ml) was achieved in 80% of children after 3 months of high-dose treatment, but only for 60% of children on 1000 IU/day of ergocalciferol [120]. A study comparing a daily equivalent dose of 3000–3500 IU of cholecalciferol (given as daily, weekly, or monthly regimens) in a cohort of children with CKD G3–4 reported that 78% of children achieved 25(OH)D ≥ 75 nmol/l (>30 ng/ml), and after switching to 1000 IU/day, 65% maintained the target after 3 months, 70% after 6 months and 82% after 9 months [127]. Further, there are two randomized dose-comparison studies (Supplementary Table S5) [186, 187]. Nadeem *et al*. randomized a cohort of children with CKD G3–5 to 1000 or 4000 IU/day and concluded that 1000 IU/day cholecalciferol is unlikely to achieve or maintain serum 25(OH)D ≥ 75 nmol/l (>30 ng/ml) [186]. The second study found that doses ≤2000 IU/day for 4 months in children with very low 25(OH)D levels (median 12.5 nmol/l, or 5 ng/ml) failed to achieve the target 25(OH)D range [187]. Noticeably, these studies showed wide variation in responses, and in the only population pharmacokinetic modeling study of cholecalciferol in children with CKD, it was suggested that optimal dosing should take into consideration body weight, baseline 25(OH)D concentration, and the presence of proteinuria [181].

The *dosing interval* varies considerable between trials, ranging from a single dose, daily, twice weekly, thrice weekly, weekly, fortnightly, to every 3, 4, 8, and 12 weeks [131]. Two studies that directly compared different dosing intervals reported comparable increase in serum 25(OH)D (Table 7) [127, 188]. Thus, similar dosages administered at different dosing intervals seem equally efficacious at increasing serum 25(OH)D levels.

**Table 7:** Randomized trials of nutritional vitamin D comparing different dosing intervals in patients with chronic kidney disease.

Study	*N*	Population	CKD stage	Vitamin D compound	Route	Intervention	Follow-up	Outcome
Iyengar 2022 [127]	90	Children < 18 years	G3–4	Chol	Oral	3000 IU/day or 25 000 IU/week or 100 000 IU/month for 3 months	3 months	Similar effects on 25(OH)D
Nata 2022 [207]	50	Adults ≥ 18 years	G5D (PD)	Ergo	Oral	20 000 IU 3×/week or 60 000 IU/week for 8 weeks	8 weeks	Similar effects on 25(OH)D

Abbreviations: 25(OH)D = 25-hydroxyvitamin-D; Chol = cholecalciferol; Ergo = ergocalciferol; PD = peritoneal dialysis; IU = international unit.

Extending the dosing interval is often accompanied by using higher doses, sometimes described as *mega-doses*, although there is currently no consensus on the definition. RCTs in community-dwelling adults, usually the elderly, have reported a higher risk of falls and fractures in those who received very high doses of oral cholecalciferol [189, 190], although a dose–response effect is unclear (Supplementary Table S6). Similar findings have not been reported in adults with CKD [108], but very few trials in CKD have used such high doses, and CKD trials are generally of short duration.

### 5.3 Use of (extended release) calcifediol in CKD

#### Clinical practice points

- Calcifediol may be considered instead of cholecalciferol or ergocalciferol in patients with CKD and liver failure.
- Extended release calcifediol may be considered in adults with CKD G3–4 and secondary hyperparathyroidism.

#### Background and rationale

There are some recent data on the use of calcifediol in adult patients with CKD (Table 8) [116, 117, 159, 191]. Calcifediol is more hydrophilic, has a shorter half-life, and does not require hepatic 25-hydroxylation for its activation. It also has a more linear and predictable dose–response relationship and increases serum 25(OH)D levels more rapidly [192, 193] than ergocalciferol or cholecalciferol. In a placebo-controlled RCT, each microgram of orally consumed calcifediol was about 5 times more effective in raising serum 25(OH)D in healthy older adults in winter than an equivalent amount of cholecalciferol [194]. An *extended-release* calcifediol product, approved by the US Food and Drug Administration and the European Medicines Agency, is available in some countries, where it is registered for the treatment of secondary hyperparathyroidism in adults with CKD G3–4 and serum total 25(OH)D < 75 nmol/l (<30 ng/ml). In trials investigating the use of extended release calcifediol in patients with CKD, the dosages utilized (30–90 μg/day) resulted in very high 25(OH)D levels, with successive PTH reduction until 25(OH)D levels exceeded 125 nmol/l (50 ng/ml) without any apparent toxicity [116, 117]. A few real-world observational studies are also available, reporting effectiveness of calcifediol in increasing serum 25(OH)D and reducing PTH without notable impact on serum calcium and phosphate [195, 196]. In a study directly comparing extended-release calcifediol to high-dose cholecalciferol and active vitamin D with low-dose cholecalciferol, extended-release calcifediol resulted in markedly higher 25(OH)D levels, with the target 25(OH)D > 75 nmol/l reached for 100% of patients treated with extended-release calcifediol, 44% treated with cholecalciferol, and 15% treated with paricalcitol and low-dose cholecalciferol [118]. Thus, extended-release calcifediol may be considered when treating vitamin D deficiency in CKD, although it should be noted that the cost of this formulation is currently considerably higher than for ergocalciferol or cholecalciferol.

**Table 8:** Randomized controlled trials investigating oral calcifediol in adults with chronic kidney disease (CKD; divide by 2.5 to convert from nmol/l to ng/ml).

Study	*N*	CKD stage	Supplement and dose	Follow-up	Baseline 25(OH)D (nmol/l)	Follow-up 25(OH)D (nmol/l)	Outcome
Morrone 2022 [191]	284	G5D (HD)	Calcifediol 40 μg ×3/week versus none	24 month	30/32	Not given	A greater proportion of patients achieved 25(OH)D>75 nmol/l: 36% versus 11%. No significant effects on mortality or cardiovascular outcomes
Levin 2017 [159]	80	G3–4	Calcifediol 5000 IU ×3/week versus placebo	6 month	63/74	235/65	Mean 25(OH)D change in the calcifediol group was 171 (145; 197) nmol/l. Pulse wave velocity decreased compared to placebo group
Sprague 2016 [116] & Strugnell 2019 [119]	144 + 285	G3–4	ERC 30–60 μg/day versus placebo	26 weeks	50/50	125–172/50	Steady-state 25(OH)D was reached after 12 weeks and averaged 125 and 140 nmol/l (30 µg/day) and 173 and 168 nmol/l (60 µg/day). Target 25(OH)D (>75 nmol/l) was achieved in 80% and 83% versus 3% and 7% (active versus placebo)
Sprague 2014 [117]	78	G3–4	ERC 30, 60, or 90 μg/day versus placebo	6 weeks	55/50	93–212/46	Mean 25(OH)D increased to 93±5 nmol/l (30 μg/day), 167 ± 44 nmol/l (60 μg/day) and 212 ± 14 nmol/l (90 μg/day). Target 25(OH)D (>75 nmol/l) was achieved in 90% versus 3% (active versus placebo)
Strugnell 2023 [118]	69	G3–4	ERC 60 μg/day versus IRC 266 μg/month versus CHL 300 000 IU/month versus PAR 1–2 μg + CHL 800 IU/day	8 weeks	52	ERC 207/IRC 63/CHL 72/PAR 60	Target 25(OH)D (>75 nmol/l) was achieved in 100% (ERC), 27% (IRC), 44% (high-dose CHL), and 15% (PAR)

Abbreviations: 25(OH)D = 25-hydroxyvitamin-D; ERC = extended release; IRC = intermediate release; IU = international unit; PAR = paricalcitol; PTH = parathyroid hormone.

### 5.4 Safety considerations

#### Key evidence points

- Hypercalcemia is rarely seen with nutritional vitamin D supplements when 25(OH)D levels are below 250 nmol/l (<100 ng/ml).
- The upper safe limit of 25(OH)D and the safety of long-term use of nutritional vitamin D in patients with CKD are unknown.

#### Clinical practice points

- In adults and children with CKD or after kidney transplantation, we suggest withholding nutritional vitamin D supplements when serum 25(OH)D levels are above 150–200 nmol/l (60–80 ng/ml) in the absence of hypercalcemia.
- In patients with CKD and hypercalcemia, check for and manage other causes of high calcium including iatrogenic (the use of active vitamin D compounds, high dialysate calcium, oral calcium-containing medications), CKD-related (presence of tertiary hyperparathyroidism), and non-CKD-related conditions (malignancy, haematological conditions, sarcoidosis, hypovitaminosis A, etc.) before stopping vitamin D supplementation.

#### Background and rationale

In the general population, large epidemiological studies have suggested a reverse J-shaped association between 25(OH)D levels and increased all-cause and cardiovascular mortality, with an increased risk at 25(OH)D  > 120 nmol/l (>48 ng/ml) [197–199]. A similar safety signal has not been reported in studies in CKD; however, although these studies indicate a linear reduction in all-cause mortality with increasing levels of 25(OH)D, their analyses have not examined 25(OH)D levels above 100 nmol/l [121, 200, 122]. Keeping in mind the substrate dependency of vitamin D metabolism in advanced CKD, observational data in children [120] and adults [29] suggest that 25OHD > 100 nmol/l (>40 ng/ml) are needed to achieve near-normal 1,25(OH)_2_D levels in CKD, which could translate to a higher safe upper limit of 25(OH)D. Concurrently, in a recent meta-analysis there was no increased risk of hypercalcemia or hyperphosphatemia in studies of nutritional vitamin D in patients with CKD [131]. Thus, vitamin D supplements are unlikely to cause hypercalcemia unless 25(OH)D levels are substantially elevated, or there is inappropriate extrarenal *CYP27B1* activity with a high degree of substrate dependence.

A particular situation arises in the post-transplant setting, where 10–20% of patients present with mild hypercalcemia, with little spontaneous remission over time [201]. Unfortunately, trials of vitamin D supplementation post-transplant have so far excluded patients with hypercalcemia [139, 140]. In *primary* hyperparathyroidism, vitamin D supplementation given to patients with hypercalcemia has been shown to decrease PTH levels prior to a parathyroidectomy, and to alleviate bone loss both before and after surgery, without meaningful increases in serum or urinary calcium [202]. Similar studies should be considered in the post-transplant setting. Currently, significant heterogeneity in investigator-defined thresholds for these biochemical measures, analytical issues, and short-duration of trials make it impossible to determine the upper safety limit and the safety of long-term use of nutritional vitamin D in patients with CKD.

## RESULTS OF THE DELPHI SURVEY

The Delphi survey revealed an overall high level of agreement. All but three clinical practice points reached the pre-defined goal of agreement, defined as being rated ‘Strongly agree’ or ‘Agree’ by more than 70% of the panel. Agreement was generally in the range of 80–90%, and disagreement (‘Disagree’ or ‘Strongly disagree’) did not exceed 10% for any of the practice points. The three practice points that failed to reach a 70% agreement received high ratings for ‘Neutral’ (27–33%), which may reflect hesitancy due to lack of conclusive evidence. Following discussions of the results of the Delphi survey, one practice point was removed (4.1), one was expanded upon in the discussion (5.1), and the last was modified for clarity (5.3). The full list of scores from the survey is available as Supplementary Table S7. All comments given in the Delphi survey were likewise reviewed by the core writing team, with subsequent minor adjustments to the clinical practice points and discussion. Many responses reflected personal practice with notable variations between responders, further highlighting the need for an evidence-informed consensus.

## RESEARCH RECOMMENDATIONS

- Data comparing vitamin D status of patients with CKD to the general population should be sought, to define whether CKD per se is a risk factor of vitamin D insufficiency.
- Research is needed to evaluate whether profiling of vitamin D metabolites beyond 25(OH)D will improve risk stratification and provide therapeutic guidance
- The optimal frequency of 25(OH)D measurement should be investigated, taking into consideration different supplementation strategies (for example cholecaliferol versus extended-release calcifediol and different dosing regimens)
- (Pragmatic) trials are needed to evaluate the effect of nutritional vitamin D supplementation on meaningful skeletal and non-skeletal (particularly cardiovascular) outcomes across different stages of CKD and after kidney transplantation.
- Future trials should primarily include participants with clinically apparent vitamin D deficiency.

## CONCLUDING REMARKS

Despite consistent associations between vitamin D deficiency and morbidity and mortality both in the general population and in patients with CKD, there is no conclusive evidence for a benefit of vitamin D supplementation on patient-relevant skeletal or non-skeletal outcomes. However, CKD is a setting of severely affected vitamin D metabolism, which contributes to the complex mineral metabolism disturbances of CKD-MBD, and vitamin D supplementation has been shown to improve the control of secondary hyperparathyroidism. Therefore, based on available evidence, we recommend monitoring for, and treating, vitamin D deficiency in adults and children with CKD across stages of disease and after kidney transplantation. Vitamin D supplementation is well-tolerated without prominent safety signals in CKD, but based on concerns raised in the background population, we suggest avoiding mega-doses of vitamin D and very high serum levels of 25(OH)D. Future research should focus on (pragmatic) clinical trials investigating the benefit of vitamin D supplementation on patient-relevant outcomes in the setting of vitamin D deficiency, across the spectrum of CKD.

## Supplementary Material

gfae293_Supplemental_File

## Data Availability

The data underlying this article are available in the article and in its online supplementary material.

## References

[1] J-DAVID Investigators , Shoji T, Inaba M, Fukagawa M et al. Effect of oral alfacalcidol on clinical outcomes in patients without secondary hyperparathyroidism receiving maintenance hemodialysis: the J-DAVID randomized clinical trial. JAMA 2018;320:2325–34. 10.1001/jama.2018.17749 30535217 PMC6583075

[2] Thadhani R, Appelbaum E, Pritchett Y et al. Vitamin D therapy and cardiac structure and function in patients with chronic kidney disease: the PRIMO randomized controlled trial. JAMA 2012;307:674–84. 10.1001/jama.2012.120 22337679

[3] Wang AY-M, Fang F, Chan J et al. Effect of paricalcitol on left ventricular mass and function in CKD—the OPERA trial. J Am Soc Nephrol 2014;25:175–86. 10.1681/ASN.2013010103 24052631 PMC3871774

[4] Ketteler M, Block GA, Evenepoel P et al. Executive summary of the 2017 KDIGO Chronic Kidney Disease–Mineral and Bone Disorder (CKD-MBD) Guideline Update: what's changed and why it matters. Kidney Int 2017;92:26–36. 10.1016/j.kint.2017.04.006 28646995

[5] Shroff R, Wan M, Nagler EV et al. Clinical practice recommendations for treatment with active vitamin D analogues in children with chronic kidney disease stages 2–5 and on dialysis. Nephrol Dial Transplant 2017;32:1114–27. 10.1093/ndt/gfx080 28873971 PMC5837664

[6] Leboff MS, Chou SH, Ratliff KA et al. Supplemental vitamin D and incident fractures in midlife and older adults. N Engl J Med 2022;387:299–309. 10.1056/NEJMoa2202106 35939577 PMC9716639

[7] Leboff MS, Chou SH, Murata EM et al. Effects of supplemental vitamin D on bone health outcomes in women and men in the VITamin D and OmegA-3 TriaL (VITAL). J Bone Miner Res 2020;35:883–93. 10.1002/jbmr.3958 31923341 PMC7217747

[8] Giustina A, Bilezikian JP, Adler RA et al. Consensus statement on vitamin D status assessment and supplementation: whys, whens, and hows. Endocr Rev 2024; 45:625–54. 10.1210/endrev/bnae009 38676447 PMC11405507

[9] Cummings SR, Rosen C. VITAL findings—a decisive verdict on vitamin D supplementation. N Engl J Med 2022;387:368–70. 10.1056/NEJMe2205993 35939583

[10] Bouillon R, LeBoff MS, Neale RE. Health effects of vitamin D supplementation: lessons learned from randomized controlled trials and mendelian randomization studies. J Bone Miner Res 2023; 38:1391–403. 10.1002/jbmr.4888 37483080 PMC10592274

[11] Bouillon R, Manousaki D, Rosen C et al. The health effects of vitamin D supplementation: evidence from human studies. Nat Rev Endocrinol 2022;18:96–110. 10.1038/s41574-021-00593-z 34815552 PMC8609267

[12] Charoenngam N, Shirvani A, Holick MF. The ongoing D-lemma of vitamin D supplementation for nonskeletal health and bone health. Curr Opin Endocrinol Diabetes Obes 2019;26:301–5. 10.1097/MED.0000000000000508 31644469

[13] Vervloet MG, Hsu S, de Boer IH. Vitamin D supplementation in people with chronic kidney disease. Kidney Int 2023; 104:698–706. 10.1016/j.kint.2023.07.010 37541585

[14] Cavalier E, Makris K, Heijboer AC et al. Vitamin D: analytical advances, clinical impact, and ongoing debates on health perspectives. Clin Chem 2024; 70:1104–21. 10.1093/clinchem/hvae056 38712647

[15] Melamed ML, Chonchol M, Gutiérrez OM et al. The role of vitamin D in CKD stages 3 to 4: report of a scientific workshop sponsored by the National Kidney Foundation. Am J Kidney Dis 2018;72:834–45. 10.1053/j.ajkd.2018.06.031 30297082 PMC6615058

[16] Bouillon R. Comparative analysis of nutritional guidelines for vitamin D. Nat Rev Endocrinol 2017;13:466–79. 10.1038/nrendo.2017.31 28387318

[17] Freisling H, Fahey MT, Moskal A et al. Region-specific nutrient intake patterns exhibit a geographical gradient within and between European countries. J Nutr 2010;140:1280–6. 10.3945/jn.110.121152 20484545

[18] Bouillon R, Bikle D. Vitamin D metabolism revised: fall of dogmas. J Bone Miner Res 2019;34:1985–92. 10.1002/jbmr.3884 31589774 PMC9000993

[19] Roizen JD, Long C, Casella A et al. Obesity decreases hepatic 25-hydroxylase activity causing low serum 25-hydroxyvitamin D. J Bone Miner Res 2019;34:1068–73 [doi]. 10.1002/jbmr.3686 30790351 PMC6663580

[20] Aatsinki S-M, Elkhwanky M-S, Kummu O et al. Fasting-induced transcription factors repress vitamin D bioactivation, a mechanism for vitamin D deficiency in diabetes. Diabetes 2019;68:918–31. 10.2337/db18-1050 30833469 PMC6477896

[21] Bikle DD, Patzek S, Wang Y. Physiologic and pathophysiologic roles of extra renal CYP27b1: case report and review. Bone Reports 2018;8:255–67. 10.1016/j.bonr.2018.02.004 29963603 PMC6021194

[22] Shimada T, Hasegawa H, Yamazaki Y et al. FGF-23 is a potent regulator of vitamin D metabolism and phosphate homeostasis. J Bone Miner Res 2004;19:429–35. 10.1359/JBMR.0301264 15040831

[23] Viaene L, Evenepoel P, Meijers B et al. Uremia suppresses immune signal-induced CYP27B1 expression in human monocytes. Am J Nephrol 2012;36:497–508. 10.1159/000345146 23171504

[24] Bacchetta J, Sea JL, Chun RF et al. Fibroblast growth factor 23 inhibits extrarenal synthesis of 1,25-dihydroxyvitamin D in human monocytes. J Bone Miner Res 2013;28:46–55. 10.1002/jbmr.1740 22886720 PMC3511915

[25] Somjen D, Weisman Y, Kohen F et al. 25-hydroxyvitamin D3-1alpha-hydroxylase is expressed in human vascular smooth muscle cells and is upregulated by parathyroid hormone and estrogenic compounds. Circulation 2005;111:1666–71. 10.1161/01.CIR.0000160353.27927.70 15795327

[26] Kanemoto Y, Hayakawa A, Sawada T et al. Transcriptional regulation of 25-hydroxyvitamin D-24-hydroxylase (CYP24A1) by calcemic factors in keratinocytes. J Nutr Sci Vitaminol (Tokyo) 2021;67:424–8. 10.3177/jnsv.67.424 34980721

[27] Meyer MB, Lee SM, Carlson AH et al. A chromatin-based mechanism controls differential regulation of the cytochrome P450 gene Cyp24a1 in renal and non-renal tissues. J Biol Chem 2019;294:14467–81. 10.1074/jbc.RA119.010173 31439663 PMC6768633

[28] Armbrecht HJ, Hodam TL, Boltz MA et al. Induction of the vitamin D 24-hydroxylase (CYP24) by 1,25-dihydroxyvitamin D3 is regulated by parathyroid hormone in UMR106 osteoblastic cells. Endocrinology 1998;139:3375–81. 10.1210/endo.139.8.6134 9681485

[29] Jørgensen HS, De Loor H, Billen J et al. Vitamin D metabolites before and after kidney transplantation in patients who are anephric. Am J Kidney Dis 2024;84:427–436.e1. 10.1053/j.ajkd.2024.03.025 38796137

[30] Stoffels K, Overbergh L, Giulietti A et al. Immune regulation of 25-hydroxyvitamin-D3-1alpha-hydroxylase in human monocytes. J Bone Miner Res 2006;21:37–47. 10.1359/JBMR.050908 16355272

[31] Bikle DD. The free hormone hypothesis: when, why, and how to measure the free hormone levels to assess vitamin D, thyroid, sex hormone, and cortisol status. JBMR Plus 2021;5:e10418. 10.1002/jbm4.10418 33553985 PMC7839820

[32] Christakos S, Dhawan P, Verstuyf A et al. Vitamin D: metabolism, molecular mechanism of action, and pleiotropic effects. Physiol Rev 2016;96:365–408. 10.1152/physrev.00014.2015 26681795 PMC4839493

[33] Pike JW, Meyer MB. The unsettled science of nonrenal calcitriol production and its clinical relevance. J Clin Invest 2020;130:4519–21. 10.1172/JCI141334 32716362 PMC7456237

[34] Meyer MB, Benkusky NA, Kaufmann M et al. Targeted genomic deletions identify diverse enhancer functions and generate a kidney-specific, endocrine-deficient Cyp27b1 pseudo-null mouse. J Biol Chem 2019;294:9518–35. 10.1074/jbc.RA119.008760 31053643 PMC6579472

[35] Dusso A, Brown A, Slatopolsky E. Extrarenal production of calcitriol. Semin Nephrol 1994;14:144–55. 8177981

[36] Braegger C, Campoy C, Colomb V et al. Vitamin D in the healthy European paediatric population. J Pediatr Gastroenterol Nutr 2013;56:692–701. 10.1097/MPG.0b013e31828f3c05 23708639

[37] Feldman D, Pike JW. Feldman and Pike's Vitamin D. 5th edition edn. Elsevier, 2023.

[38] Herrick KA, Storandt RJ, Afful J et al. Vitamin D status in the United States, 2011–2014. Am J Clin Nutr 2019;110:150–7. 10.1093/ajcn/nqz037 31076739 PMC7263437

[39] Bouillon R, Carmeliet G. Vitamin D insufficiency: definition, diagnosis and management. Best Pract Res Clin Endocrinol Metab 2018;32:669–84. 10.1016/j.beem.2018.09.014 30449548

[40] Munns CF, Shaw N, Kiely M et al. Global consensus recommendations on prevention and management of nutritional rickets. J Clin Endocrinol Metab 2016;101:394–415. 10.1210/jc.2015-2175 26745253 PMC4880117

[41] Need AG, O'loughlin PD, Morris HA et al. Vitamin D metabolites and calcium absorption in severe vitamin D deficiency. J Bone Miner Res 2008;23:1859–63. 10.1359/jbmr.080607 18597633

[42] Lips P, Duong T, Oleksik A et al. A global study of vitamin D status and parathyroid function in postmenopausal women with osteoporosis: baseline data from the multiple outcomes of raloxifene evaluation clinical trial. J Clin Endocrinol Metab 2001;86:1212–21. 10.1210/jcem.86.3.7327 11238511

[43] Malabanan A, Veronikis IE, Holick MF. Redefining vitamin D insufficiency. Lancet 1998;351:805–6. 10.1016/S0140-6736(05)78933-9 9519960

[44] Levin A, Bakris GL, Molitch M et al. Prevalence of abnormal serum vitamin D, PTH, calcium, and phosphorus in patients with chronic kidney disease: results of the study to evaluate early kidney disease. Kidney Int 2007;71:31–38. 10.1038/sj.ki.5002009 17091124

[45] Wolf M, Shah A, Gutierrez O et al. Vitamin D levels and early mortality among incident hemodialysis patients. Kidney Int 2007;72:1004–13. 10.1038/sj.ki.5002451 17687259

[46] Ureña-Torres P, Metzger M, Haymann JP et al. Association of kidney function, vitamin D deficiency, and circulating markers of mineral and bone disorders in CKD. Am J Kidney Dis 2011;58:544–53. 10.1053/j.ajkd.2011.04.029 21803465

[47] Barragry JM, Carter ND, Beer M et al. Vitamin-D metabolism in nephrotic syndrome. Lancet 1977;310:629–32. 10.1016/S0140-6736(77)92498-9 71448

[48] Grymonprez A, Proesmans W, Van Dyck M et al. Vitamin D metabolites in childhood nephrotic syndrome. Pediatr Nephrol 1995;9:278–81. 10.1007/BF02254183 7632510

[49] Joffe P, Heat JG. Vitamin D and vitamin-D-binding protein kinetics in patients treated with continuous ambulatory peritoneal dialysis (CAPD). Perit Dial Int 1989;9:281–4. 10.1177/089686088900900410 2488382

[50] Prytuła A, Wells D, Mclean T et al. Urinary and dialysate losses of vitamin D-binding protein in children on chronic peritoneal dialysis. Pediatr Nephrol 2012;27:643–9. 10.1007/s00467-011-2045-0 22081234

[51] Michaud J, Naud J, Ouimet D et al. Reduced hepatic synthesis of calcidiol in uremia. J Am Soc Nephrol 2010;21:1488–97. 10.1681/ASN.2009080815 20595682 PMC3013518

[52] Cani PD, Amar J, Iglesias MA et al. Metabolic endotoxemia initiates obesity and insulin resistance. Diabetes 2007;56:1761–72. 10.2337/db06-1491 17456850

[53] Evenepoel P, Stenvinkel P, Shanahan C et al. Inflammation and gut dysbiosis as drivers of CKD-MBD. Nat Rev Nephrol 2023;19:646–57. 10.1038/s41581-023-00736-7 37488276

[54] Mithal A, Wahl DA, Bonjour J-P et al. Global vitamin D status and determinants of hypovitaminosis D. Osteoporos Int 2009;20:1807–20. 10.1007/s00198-009-0954-6 19543765

[55] Tobias DK, Luttmann-Gibson H, Mora S et al. Association of body weight with response to vitamin D supplementation and metabolism. JAMA Netw Open 2023;6:e2250681. 10.1001/jamanetworkopen.2022.50681 36648947 PMC9856931

[56] Kim SM, Choi HJ, Lee JP et al. Prevalence of vitamin D deficiency and effects of supplementation with cholecalciferol in patients with chronic kidney disease. J Ren Nutr 2014;24:20–25. 10.1053/j.jrn.2013.07.003 24216258

[57] Hasegawa H, Nagano N, Urakawa I et al. Direct evidence for a causative role of FGF23 in the abnormal renal phosphate handling and vitamin D metabolism in rats with early-stage chronic kidney disease. Kidney Int 2010;78:975–80. 10.1038/ki.2010.313 20844473

[58] Shalhoub V, Shatzen EM, Ward SC et al. FGF23 neutralization improves chronic kidney disease-associated hyperparathyroidism yet increases mortality. J Clin Invest 2012;122:2543–53. 10.1172/JCI61405 22728934 PMC3386816

[59] Bosworth C, de Boer IH. Impaired vitamin D metabolism in CKD. Semin Nephrol 2013;33:158–68. 10.1016/j.semnephrol.2012.12.016 23465502 PMC3592201

[60] Gallieni M, Kamimura S, Ahmed A et al. Kinetics of monocyte 1 alpha-hydroxylase in renal failure. Am J Physiol Renal Physiol 1995;268:F746–53. 10.1152/ajprenal.1995.268.4.F746 7733332

[61] Glorieux G, Vanholder R. Blunted response to vitamin D in uremia. Kidney Int Suppl 2001;78:S182–185. 10.1046/j.1523-1755.2001.59780182.x 11169007

[62] Sawaya BP, Koszewski NJ, Qi Q et al. Secondary hyperparathyroidism and vitamin D receptor binding to vitamin D response elements in rats with incipient renal failure. J Am Soc Nephrol 1997;8:271–8. 10.1681/ASN.V82271 9048346

[63] Perrin P, Caillard S, Javier RM et al. Persistent hyperparathyroidism is a major risk factor for fractures in the five years after kidney transplantation. Am J Transplant 2013;13:2653–63. 10.1111/ajt.12425 24034142

[64] Torres A, Torregrosa V, Marcen R et al. Mineral metabolism disorders, vertebral fractures and aortic calcifications in stable kidney transplant recipients: the role of gender (EMITRAL study). Nefrología 2016;36:255–67. 10.1016/j.nefro.2016.03.004 27133898

[65] Yin S, Wang X, Li L et al. Prevalence of vitamin D deficiency and impact on clinical outcomes after kidney transplantation: a systematic review and meta-analysis. Nutr Rev 2022;80:950–61. 10.1093/nutrit/nuab058 34472620

[66] Perrin P, Kiener C, Javier R-M et al. Recent changes in chronic kidney disease–mineral and bone disorders and associated fractures after kidney transplantation. Transplantation 2017;101:1897–905. 10.1097/TP.0000000000001449 27547867

[67] Evenepoel P, Naesens M, Claes K et al. Tertiary ‘hyperphosphatoninism’ accentuates hypophosphatemia and suppresses calcitriol levels in renal transplant recipients. Am J Transplant 2007;7:1193–200. 10.1111/j.1600-6143.2007.01753.x 17359508

[68] Evenepoel P, Meijers BKI, De Jonge H et al. Recovery of hyperphosphatoninism and renal phosphorus wasting one year after successful renal transplantation. Clin J Am Soc Nephrol 2008;3:1829–36. 10.2215/CJN.01310308 18922992 PMC2572285

[69] Wolf M, Weir MR, Kopyt N et al. A prospective cohort study of mineral metabolism after kidney transplantation. Transplantation 2016;100:184–93. 10.1097/TP.0000000000000823 26177089 PMC4683035

[70] Cavalier E, Lukas, P, Beaert, A-C et al. Analytical and clinical evaluation of the new Fujirebio Lumipulse(R)G non-competitive assay for 25(OH)-vitamin D and three immunoassays for 25(OH)D in healthy subjects, osteoporotic patients, third trimester pregnant women, healthy African subjects, hemodialyzed and intensive care patients. Clin Chem Lab Med 2016;54:1347–55. 26741345 10.1515/cclm-2015-0923

[71] Depreter B, Heijboer AC, Langlois MR. Accuracy of three automated 25-hydroxyvitamin D assays in hemodialysis patients. Clin Chim Acta 2013;415:255–60. 10.1016/j.cca.2012.10.056 23159781

[72] Hsu S, Zelnick LR, Lin YS et al. Validation of the 24,25-dihydroxyvitamin D(3) to 25-hydroxyvitamin D(3) ratio as a biomarker of 25-hydroxyvitamin D(3) clearance. J Steroid Biochem Mol Biol 2022;217:106047. 10.1016/j.jsbmb.2021.106047 34954017 PMC8837693

[73] Ginsberg C, Hoofnagle AN, Katz R et al. The vitamin D metabolite ratio is independent of vitamin D binding protein concentration. Clin Chem 2021;67:385–93. 10.1093/clinchem/hvaa238 33188595 PMC8880257

[74] De Boer IH, Sachs MC, Chonchol M et al. Estimated GFR and circulating 24,25-dihydroxyvitamin D3 concentration: a participant-level analysis of 5 cohort studies and clinical trials. Am J Kidney Dis 2014;64:187–97. 10.1053/j.ajkd.2014.02.015 24703961 PMC4111986

[75] Bosworth CR, Levin G, Robinson-Cohen C et al. The serum 24,25-dihydroxyvitamin D concentration, a marker of vitamin D catabolism, is reduced in chronic kidney disease. Kidney Int 2012;82:693–700. 10.1038/ki.2012.193 22648296 PMC3434313

[76] Graeff-Armas LA, Kaufmann M, Lyden E et al. Serum 24,25-dihydroxyvitamin D(3) response to native vitamin D(2) and D(3) supplementation in patients with chronic kidney disease on hemodialysis. Clin Nutr 2018;37:1041–5. 10.1016/j.clnu.2017.04.020 28506446

[77] Stubbs JR, Zhang S, Friedman PA et al. Decreased conversion of 25-hydroxyvitamin D 3 to 24,25-dihydroxyvitamin D 3 following cholecalciferol therapy in patients with CKD. Clin J Am Soc Nephrol 2014;9:1965–73. 10.2215/CJN.03130314 25183657 PMC4220759

[78] Lee S, Chung HJ, Jung S et al. 24,25-Dihydroxy vitamin D and vitamin D metabolite ratio as biomarkers of vitamin D in chronic kidney disease. Nutrients 2023; 15:578. 10.3390/nu15030578 36771287 PMC9920774

[79] Hsu S, Zelnick LR, Lin YS et al. Differences in 25-hydroxyvitamin D clearance by eGFR and race: a pharmacokinetic study. J Am Soc Nephrol 2021;32:188–98. 10.1681/ASN.2020050625 33115916 PMC7894669

[80] Hsu S, Zelnick LR, Bansal N et al. Vitamin D metabolites and risk of cardiovascular disease in chronic kidney disease: the CRIC study. J Am Heart Assoc 2023;12:e028561. 10.1161/JAHA.122.028561 37421259 PMC10382125

[81] Bikle DD, Schwartz J. Vitamin D binding protein, total and free vitamin D levels in different physiological and pathophysiological conditions. Front Endocrinol 2019;10:317. 10.3389/fendo.2019.00317 PMC6546814 31191450

[82] Vermeulen A, Verdonck L, Kaufman JM. A critical evaluation of simple methods for the estimation of free testosterone in serum. J Clin Endocrinol Metab 1999;84:3666–72. 10.1210/jcem.84.10.6079 10523012

[83] Henderson CM, Lutsey PL, Misialek JR et al. Measurement by a novel LC-MS/MS methodology reveals similar serum concentrations of vitamin D-binding protein in blacks and whites. Clin Chem 2016;62:179–87. 10.1373/clinchem.2015.244541 26453697 PMC4698095

[84] Heureux N, Lindhout E, Swinkels L. A direct assay for measuring free 25-hydroxyvitamin D. J AOAC Int 2017;100:1318–22. 10.5740/jaoacint.17-0084 28492143

[85] Ishimine N, Wu S, Ota R et al. Development of free 25-hydroxyvitamin D3 assay method using liquid chromatography–tandem mass spectrometry. Biosci Rep 2022;42. 10.1042/BSR20221326 PMC9547169 36107130

[86] Van Hoof H, De Sevaux R, Van Baelen H et al. Relationship between free and total 1,25-dihydroxyvitamin D in conditions of modified binding. Eur J Endocrinol 2001;144:391–6. 10.1530/eje.0.1440391 11275949

[87] Chen X, Lu Y-P, Luo T et al. Free 25-vitamin D is correlated with cardiovascular events in prevalent hemodialysis patients but not with markers of renal mineral bone disease. Kidney Blood Press Res 2019;44:344–53. 10.1159/000499878 31203281

[88] Zeng S, Bachert D, Pavkovic M et al. Free vitamin D is independently associated with systolic blood pressure in diabetic patients with impaired kidney function. Clin Nephrol 2022;97:63–69. 10.5414/CN110549 34779388

[89] Ennis JL, Worcester EM, Coe FL et al. Current recommended 25-hydroxyvitamin D targets for chronic kidney disease management may be too low. J Nephrol 2016;29:63–70. 10.1007/s40620-015-0186-0 25736620

[90] Dhillon-Jhattu S, Mcgill RL, Ennis JL et al. Vitamin D and parathyroid hormone levels in CKD. Am J Kidney Dis 2023;81:122–4. 10.1053/j.ajkd.2022.06.006 35926776

[91] Christodoulou M, Aspray TJ, Schoenmakers I. Vitamin D supplementation for patients with chronic kidney disease: a systematic review and meta-analyses of trials investigating the response to supplementation and an overview of guidelines. Calcif Tissue Int 2021;109:157–78. 10.1007/s00223-021-00844-1 33895867 PMC8273061

[92] Alvarez JA, Law J, Coakley KE et al. High-dose cholecalciferol reduces parathyroid hormone in patients with early chronic kidney disease: a pilot, randomized, double-blind, placebo-controlled trial. Am J Clin Nutr 2012;96:672–9. 10.3945/ajcn.112.040642 22854402 PMC3417221

[93] Gravesen E, Hofman-Bang J, Lewin E et al. Ergocalciferol treatment and aspects of mineral homeostasis in patients with chronic kidney disease stage 4–5. Scand J Clin Lab Invest 2013;73:107–16. 10.3109/00365513.2012.744464 23281842

[94] Petchey WG, Hickman IJ, Prins JB et al. Vitamin D does not improve the metabolic health of patients with chronic kidney disease stage 3–4: a randomized controlled trial. Nephrology 2013;18:26–35. 10.1111/j.1440-1797.2012.01662.x 23043683

[95] Marckmann P, Agerskov H, Thineshkumar S et al. Randomized controlled trial of cholecalciferol supplementation in chronic kidney disease patients with hypovitaminosis D. Nephrol Dial Transplant 2012;27:3523–31. 10.1093/ndt/gfs138 22822092

[96] Westerberg P-A, Sterner G, Ljunggren Ö et al. High doses of cholecalciferol alleviate the progression of hyperparathyroidism in patients with CKD stages 3–4: results of a 12-week double-blind, randomized, controlled study. Nephrol Dial Transplant 2018;33:466–71. 10.1093/ndt/gfx059 29156056 PMC6018863

[97] Yadav AK, Kumar V, Kumar V et al. The effect of vitamin D supplementation on bone metabolic markers in chronic kidney disease. J Bone Miner Res 2018;33:404–9. 10.1002/jbmr.3314 29044707

[98] Kumar V, Yadav AK, Lal A et al. A randomized trial of vitamin D supplementation on vascular function in CKD. J Am Soc Nephrol 2017;28:3100–8. 10.1681/ASN.2017010003 28667080 PMC5619968

[99] Dogan E, Erkoc R, Sayarlioglu H et al. Effect of depot oral cholecalciferol treatment on secondary hyperparathyroidism in stage 3 and stage 4 chronic kidney diseases patients. Ren Fail 2008;30:407–10. 10.1080/08860220801964210 18569914

[100] Chandra P, Binongo JNG, Ziegler TR et al. Cholecalciferol (vitamin D3) therapy and vitamin D insufficiency in patients with chronic kidney disease: a randomized controlled pilot study. Endocr Pract 2008;14:10–17. 10.4158/EP.14.1.10 18238736 PMC2654595

[101] Wetmore JB, Kimber C, Mahnken JD et al. Cholecalciferol v. ergocalciferol for 25-hydroxyvitamin D (25(OH)D) repletion in chronic kidney disease: a randomised clinical trial. Br J Nutr 2016;116:2074–81. 10.1017/S000711451600427X 28065190 PMC6036626

[102] Matuszkiewicz-Rowińska J, Kulicki P, Zebrowski P et al. Cholecalciferol vs. small doses of alfacalcidol vs. placebo in chronic kidney disease patients on hemodialysis: a randomized parallel group study. Front Med (Lausanne) 2021;8:781191. 10.3389/fmed.2021.781191 35127748 PMC8814355

[103] Brimble KS, Ganame J, Margetts P et al. Impact of bioelectrical impedance-guided fluid management and vitamin D supplementation on left ventricular mass in patients receiving peritoneal dialysis: a randomized controlled trial. Am J Kidney Dis 2022;79:820–31. 10.1053/j.ajkd.2021.08.022 34656640

[104] Alshahawey M, El Borolossy R, El Wakeel L et al. The impact of cholecalciferol on markers of vascular calcification in hemodialysis patients: a randomized placebo controlled study. Nutr Metab Cardiovasc Dis 2021;31:626–33. 10.1016/j.numecd.2020.09.014 33594986

[105] Zheng C-M, Wu C-C, Hung C-F et al. Cholecalciferol additively reduces serum parathyroid hormone levels in severe secondary hyperparathyroidism treated with calcitriol and cinacalcet among hemodialysis patients. Nutrients 2018;10:196. 10.3390/nu10020196 29439405 PMC5852772

[106] Zheng J-Q, Hou Y-C, Zheng C-M et al. Cholecalciferol additively reduces serum parathyroid hormone and increases vitamin D and cathelicidin levels in paricalcitol-treated secondary hyperparathyroid hemodialysis patients. Nutrients 2016;708. 10.3390/nu8110708 27827962 PMC5133095

[107] Wang Y, Liu Y, Lian Y et al. Efficacy of high-dose supplementation with oral vitamin D3 on depressive symptoms in dialysis patients with vitamin D3 insufficiency: a prospective, randomized, double-blind study. J Clin Psychopharmacol 2016;36:229–35. 10.1097/JCP.0000000000000486 27022679

[108] Massart A, Debelle FD, Racapé J et al. Biochemical parameters after cholecalciferol repletion in hemodialysis: results from the VitaDial randomized trial. Am J Kidney Dis 2014;64:696–705. 10.1053/j.ajkd.2014.04.020 24856872

[109] Li L, Lin M, Krassilnikova M et al. Effect of cholecalciferol supplementation on inflammation and cellular alloimmunity in hemodialysis patients: data from a randomized controlled pilot trial. PLoS One 2014;9:e109998. 10.1371/journal.pone.0109998 25296334 PMC4190314

[110] Hewitt NA, O'Connor AA, O'Shaughnessy DV et al. Effects of cholecalciferol on functional, biochemical, vascular, and quality of life outcomes in hemodialysis patients. Clin J Am Soc Nephrol 2013;8:1143–9. 10.2215/CJN.02840312 23493381 PMC3700687

[111] Delanaye P, Weekers L, Warling X et al. Cholecalciferol in haemodialysis patients: a randomized, double-blind, proof-of-concept and safety study. Nephrol Dial Transplant 2013;28:1779–86. 10.1093/ndt/gft001 23378417

[112] Dreyer G, Tucker AT, Harwood SM et al. Ergocalciferol and microcirculatory function in chronic kidney disease and concomitant vitamin D deficiency: an exploratory, double blind, randomised controlled trial. PLoS One 2014;9:e99461. 10.1371/journal.pone.0099461 25006678 PMC4090117

[113] Bhan I, Dobens D, Tamez H et al. Nutritional vitamin D supplementation in dialysis: a randomized trial. Clin J Am Soc Nephrol 2015;10:611–9. 10.2215/CJN.06910714 25770176 PMC4386253

[114] Zhang D, Li H, Yin D et al. Ergocalciferol versus calcitriol for controlling chronic kidney disease mineral bone disorder in stage 3 to 5 CKD: a randomized controlled trial. Eur J Pharmacol 2016;789:127–33. 10.1016/j.ejphar.2016.07.019 27401037

[115] Batacchi Z, Robinson-Cohen C, Hoofnagle AN et al. Effects of vitamin D(2) supplementation on vitamin D(3) metabolism in health and CKD. Clin J Am Soc Nephrol 2017;12:1498–506. 10.2215/CJN.00530117 28768705 PMC5586563

[116] Sprague SM, Crawford PW, Melnick JZ et al. Use of extended-release calcifediol to treat secondary hyperparathyroidism in stages 3 and 4 chronic kidney disease. Am J Nephrol 2016;44:316–25. 10.1159/000450766 27676085

[117] Sprague SM, Silva AL, Al-Saghir F et al. Modified-release calcifediol effectively controls secondary hyperparathyroidism associated with vitamin D insufficiency in chronic kidney disease. Am J Nephrol 2014;40:535–45. 10.1159/000369939 25572630

[118] Strugnell SA, Csomor P, Ashfaq A et al. Evaluation of therapies for secondary hyperparathyroidism associated with vitamin D insufficiency in chronic kidney disease. Kidney Dis (Basel) 2023;9:206–17. 10.1159/000529523 37497207 PMC10368011

[119] Strugnell SA, Sprague SM, Ashfaq A et al. Rationale for raising current clinical practice guideline target for serum 25-hydroxyvitamin D in chronic kidney disease. Am J Nephrol 2019;49:284–93. 10.1159/000499187 30878999

[120] Shroff R, Wan M, Gullett A et al. Ergocalciferol supplementation in children with CKD delays the onset of secondary hyperparathyroidism: a randomized trial. Clin J Am Soc Nephrol 2012;7:216–23. 10.2215/CJN.04760511 22266572

[121] Jean G, Souberbielle JC, Chazot C. Monthly cholecalciferol administration in haemodialysis patients: a simple and efficient strategy for vitamin D supplementation. Nephrol Dial Transplant 2009;24:3799–805. 10.1093/ndt/gfp370 19622574

[122] Jean G, Terrat J-C, Vanel T et al. Daily oral 25-hydroxycholecalciferol supplementation for vitamin D deficiency in haemodialysis patients: effects on mineral metabolism and bone markers. Nephrol Dial Transplant 2008;23:3670–6. 10.1093/ndt/gfn339 18579534

[123] Matias PJ, Jorge C, Ferreira C et al. Cholecalciferol supplementation in hemodialysis patients: effects on mineral metabolism, inflammation, and cardiac dimension parameters. Clin J Am Soc Nephrol 2010;5:905–11. 10.2215/CJN.06510909 20203163 PMC2863968

[124] Huish SA, Jenkinson C, Dunn JA et al. Low serum 1,25(OH)2D3 in end-stage renal disease: is reduced 1alpha-hydroxylase the only problem? Endocr Connect 2021;10:1291–8. 10.1530/EC-21-0372 34519274 PMC8558908

[125] Shroff R, Wan M, Nagler EV et al. Clinical practice recommendations for native vitamin D therapy in children with chronic kidney disease stages 2–5 and on dialysis. Nephrol Dial Transplant 2017;32:1098–113. 10.1093/ndt/gfx065 28873969 PMC5837199

[126] Hari P, Gupta N, Hari S et al. Vitamin D insufficiency and effect of cholecalciferol in children with chronic kidney disease. Pediatr Nephrol 2010;25:2483–8. 10.1007/s00467-010-1639-2 20872152

[127] Iyengar A, Kamath N, Reddy HV et al. Determining the optimal cholecalciferol dosing regimen in children with CKD: a randomized controlled trial. Nephrol Dial Transplant 2022;37:326–34. 10.1093/ndt/gfaa369 33367869

[128] Kamath N, Iyengar A, Reddy HV et al. Changes in bone biomarkers in response to different dosing regimens of cholecalciferol supplementation in children with chronic kidney disease. Pediatr Nephrol 2023;38:1907–13. 10.1007/s00467-022-05790-0 36322258

[129] DeLuca HF. Overview of general physiologic features and functions of vitamin D. Am J Clin Nutr 2004;80:1689S–96S. 10.1093/ajcn/80.6.1689S 15585789

[130] Bhan A, Qiu S, Rao SD. Bone histomorphometry in the evaluation of osteomalacia. Bone Reports 2018;8:125–34. 10.1016/j.bonr.2018.03.005 29955631 PMC6020114

[131] Yeung W-CG, Palmer SC, Strippoli GFM et al. Vitamin D therapy in adults with CKD: a systematic review and meta-analysis. Am J Kidney Dis 2023;82:543–58. 10.1053/j.ajkd.2023.04.003 37356648

[132] Bosworth C, Boer IHD, Targher G et al. The effect of combined calcium and cholecalciferol supplementation on bone mineral density in elderly women with moderate chronic kidney disease. Clin Nephrol 2012;77:358–65. 10.5414/CN107180 22551881 PMC4030712

[133] Priemel M, Von Domarus C, Klatte TO et al. Bone mineralization defects and vitamin D deficiency: histomorphometric analysis of iliac crest bone biopsies and circulating 25-hydroxyvitamin D in 675 patients. J Bone Miner Res 2010;25:305–12. 10.1359/jbmr.090728 19594303

[134] Coen G, Mantella D, Manni M et al. 25-hydroxyvitamin D levels and bone histomorphometry in hemodialysis renal osteodystrophy. Kidney Int 2005;68:1840–8. 10.1111/j.1523-1755.2005.00603.x 16164662

[135] Hamdy NAT, Kanis JA, Beneton MNC et al. Effect of alfacalcidol on natural course of renal bone disease in mild to moderate renal failure. BMJ 1995;310:358–63. 10.1136/bmj.310.6976.358 7677827 PMC2548761

[136] Cannella G, Bonucci E, Rolla D et al. Evidence of healing of secondary hyperparathyroidism in chronically hemodialyzed uremic patients treated with long-term intravenous calcitriol. Kidney Int 1994;46:1124–32. 10.1038/ki.1994.375 7861707

[137] Heaf J, Tvedegaard E, Kanstrup I‐L et al. Bone loss after renal transplantation: role of hyperparathyroidism, acidosis, cyclosporine and systemic disease. Clin Transplant 2000;14:457–63. 10.1034/j.1399-0012.2000.140503.x 11048990

[138] Jørgensen HS, Behets G, Bammens B et al. Patterns of renal osteodystrophy 1 year after kidney transplantation. Nephrol Dial Transplant 2021;36:2130–9. 10.1093/ndt/gfab239 34383929

[139] Courbebaisse M, Bourmaud A, Souberbielle J-C et al. Nonskeletal and skeletal effects of high doses versus low doses of vitamin D(3) in renal transplant recipients: results of the VITALE (VITamin D supplementation in renAL transplant recipients) study, a randomized clinical trial. Am J Transplant 2023;23:366–76. 10.1016/j.ajt.2022.12.007 36695682

[140] Tsujita M, Doi Y, Obi Y et al. Cholecalciferol supplementation attenuates bone loss in incident kidney transplant recipients: a prespecified secondary endpoint analysis of a randomized controlled trial. J Bone Miner Res 2020;37:303–11. 10.1002/jbmr.4469 PMC9298992 34747516

[141] Courbebaisse M, Thervet E, Souberbielle JC et al. Effects of vitamin D supplementation on the calcium–phosphate balance in renal transplant patients. Kidney Int 2009;75:646–51. 10.1038/ki.2008.549 18923386

[142] Wissing KM, Broeders N, Moreno-Reyes R et al. A controlled study of vitamin D3 to prevent bone loss in renal-transplant patients receiving low doses of steroids. Transplantation 2005;79:108–15. 10.1097/01.TP.0000149322.70295.A5 15714177

[143] Talalaj M, Gradowska, L, Marcinowska-Suchowierska, E et al. Efficiency of preventive treatment of glucocorticoid-induced osteoporosis with 25-hydroxyvitamin D3 and calcium in kidney transplant patients. Transplant Proc 1996;28:3485–7. 8962355

[144] Courbebaisse M, Bourmaud A, Souberbielle J-C et al. Nonskeletal and skeletal effects of high doses versus low doses of vitamin D3 in renal transplant recipients: results of the VITALE (VITamin D supplementation in renAL transplant recipients) study, a randomized clinical trial. Am J Transplant 2023; 23:366–76. 10.1016/j.ajt.2022.12.007 36695682

[145] Pike JW, Meyer MB, Lee SM et al. The vitamin D receptor: contemporary genomic approaches reveal new basic and translational insights. J Clin Invest 2017;127:1146–54. 10.1172/JCI88887 28240603 PMC5373853

[146] Zhang R, Li B, Gao X et al. Serum 25-hydroxyvitamin D and the risk of cardiovascular disease: dose–response meta-analysis of prospective studies. Am J Clin Nutr 2017;105:810–9. 10.3945/ajcn.116.140392 28251933

[147] Herrmann M, Keppel MH, Zelzer S et al. The role of functional vitamin D deficiency and low vitamin D reservoirs in relation to cardiovascular health and mortality. Clin Chem Lab Med 2024; 63:208–19. 10.1515/cclm-2024-0391 38890759

[148] Zhang Y, Darssan D, Pascoe EM et al. Vitamin D status and mortality risk among patients on dialysis: a systematic review and meta-analysis of observational studies. Nephrol Dial Transplant 2018;33:1742–51. 10.1093/ndt/gfy016 29481620

[149] Pilz S, Iodice S, Zittermann A et al. Vitamin D status and mortality risk in CKD: a meta-analysis of prospective studies. Am J Kidney Dis 2011;58:374–82. 10.1053/j.ajkd.2011.03.020 21636193

[150] Duranton F, Rodriguez-Ortiz ME, Duny Y et al. Vitamin D treatment and mortality in chronic kidney disease: a systematic review and meta-analysis. Am J Nephrol 2013;37:239–48. 10.1159/000346846 23467111

[151] Manson JE, Cook NR, Lee I-M et al. Vitamin D supplements and prevention of cancer and cardiovascular disease. N Engl J Med 2019;380:33–44. 10.1056/NEJMoa1809944 30415629 PMC6425757

[152] Scragg R, Stewart AW, Waayer D et al. Effect of monthly high-dose vitamin D supplementation on cardiovascular disease in the vitamin D assessment study: a randomized clinical trial. JAMA Cardiol 2017;2:608–16. 10.1001/jamacardio.2017.0175 28384800 PMC5815022

[153] Trivedi DP . Effect of four monthly oral vitamin D3 (cholecalciferol) supplementation on fractures and mortality in men and women living in the community: randomised double blind controlled trial. BMJ 2003;326:469. 10.1136/bmj.326.7387.469 12609940 PMC150177

[154] Neale RE, Baxter C, Romero BD et al. The D-Health Trial: a randomised controlled trial of the effect of vitamin D on mortality. Lancet Diabetes Endocrinol 2022;10:120–8. 10.1016/S2213-8587(21)00345-4 35026158

[155] Sutherland JP, Zhou A, Hyppönen E. Vitamin D deficiency increases mortality risk in the UK Biobank: a nonlinear mendelian randomization study. Ann Intern Med 2022;175:1552–9. 10.7326/M21-3324 36279545

[156] Lips P, Wiersinga A, Van Ginkel FC et al. The effect of vitamin D supplementation on vitamin D status and parathyroid function in elderly subjects. J Clin Endocrin Metab 1988;67:644–50. 10.1210/jcem-67-4-644 3417845

[157] Limonte CP, Zelnick LR, Hoofnagle AN et al. Effects of vitamin D(3) supplementation on cardiovascular and cancer outcomes by eGFR in VITAL. Kidney360 2022;3:2095–105. 10.34067/KID.0006472022 36591342 PMC9802543

[158] NIHR, N. I. f. H. a. C. R . Survival Improvement with Cholecalciferol in Patients on Dialysis the SIMPLIFIED Registry Trial, https://fundingawards.nihr.ac.uk/award/14/49/127

[159] Levin A, Tang M, Perry T et al. Randomized controlled trial for the effect of vitamin D supplementation on vascular stiffness in CKD. Clin J Am Soc Nephrol 2017;12:1447–60. 10.2215/CJN.10791016 28550081 PMC5586581

[160] Das S, Selvarajan S, Kamalanathan S et al. A randomized double-blind placebo-controlled trial evaluating the efficacy of oral cholecalciferol in improving renal and vascular functions in vitamin D-deficient patients with type 2 diabetes mellitus. J Diet Suppl 2023;20:44–54. 10.1080/19390211.2021.1958041 34387520

[161] Samaan F, Carvalho AB, Pillar R et al. The effect of long-term cholecalciferol supplementation on vascular calcification in chronic kidney disease patients with hypovitaminosis D. J Ren Nutr 2019;29:407–15. 10.1053/j.jrn.2018.12.002 30686750

[162] Banerjee D, Chitalia N, Ster IC et al. Impact of vitamin D on cardiac structure and function in CKD patients with hypovitaminosis D, a randomised controlled trial and meta-analysis. Eur Heart J Cardiovasc Pharmacother 2019; 7:302–11. 10.1093/ehjcvp/pvz080 PMC8302255 31830258

[163] Liyanage GC, Lekamwasam S, Weerarathna TP et al. Effects of high-dose parenteral vitamin D therapy on lipid profile and blood pressure in patients with diabetic nephropathy: a randomized double-blind clinical trial. Diabetes Metab Syndr 2017;11 Suppl 2:S767–70. 10.1016/j.dsx.2017.05.013 28606441

[164] Krummel T, Ingwiller M, Keller N et al. Effects of high- vs low-dose native vitamin D on albuminuria and the renin–angiotensin–aldosterone system: a randomized pilot study. Int Urol Nephrol 2022;54:895–905. 10.1007/s11255-021-02950-3 34286472

[165] Zittermann A, Berthold HK, Pilz S. The effect of vitamin D on fibroblast growth factor 23: a systematic review and meta-analysis of randomized controlled trials. Eur J Clin Nutr 2021;75:980–7. 10.1038/s41430-020-00725-0 32855522 PMC8510890

[166] Daroux M, Shenouda M, Bacri J-L et al. Vitamin D2 versus vitamin D3 supplementation in hemodialysis patients: a comparative pilot study. J Nephrol 2013;26:152–7. 10.5301/jn.5000123 22641581

[167] Balachandar R, Pullakhandam R, Kulkarni B et al. Relative efficacy of vitamin D(2) and vitamin D(3) in improving vitamin D status: systematic review and meta-analysis. Nutrients 2021; 3:3328. 10.3390/nu13103328 PMC8538717 34684328

[168] van den Heuvel EG, Lips P, Schoonmade LJ et al. Comparison of the effect of daily vitamin D2 and vitamin D3 supplementation on serum 25-hydroxyvitamin D concentration (total 25(OH)D, 25(OH)D2, and 25(OH)D3) and importance of body mass index: a systematic review and meta-analysis. Adv Nutr 2024;15:100133. 10.1016/j.advnut.2023.09.016 37865222 PMC10831883

[169] Tripkovic L, Lambert H, Hart K et al. Comparison of vitamin D2 and vitamin D3 supplementation in raising serum 25-hydroxyvitamin D status: a systematic review and meta-analysis. Am J Clin Nutr 2012;95:1357–64. 10.3945/ajcn.111.031070 22552031 PMC3349454

[170] Behairy MA, Elsharabasy RM, El Shaarawy AB et al. Oral versus intramuscular cholecalciferol replacement in hemodialysis patients with vitamin D deficiency. J Nephropharmacol 2022;11:e07. 10.34172/npj.2022.07

[171] Gupta N, Farooqui KJ, Batra CM et al. Effect of oral versus intramuscular vitamin D replacement in apparently healthy adults with vitamin D deficiency. Indian J Endocrinol Metab 2017;21:131–6. 10.4103/2230-8210.196007 28217512 PMC5240054

[172] Tellioglu A, Basaran S, Guzel R et al. Efficacy and safety of high dose intramuscular or oral cholecalciferol in vitamin D deficient/insufficient elderly. Maturitas 2012;72:332–8. 10.1016/j.maturitas.2012.04.011 22613271

[173] Albinsson E, Grönlund AB, Paulsson M et al. Unpredictable supplementation of vitamin D to infants in the neonatal intensive care unit: an experimental study. Acta Paediatr 2024; 10.1111/apa.17351 38972986

[174] Wan M, Patel A, Patel JP et al. Quality and use of unlicensed vitamin D preparations in primary care in England: retrospective review of national prescription data and laboratory analysis. Br J Clin Pharmacol 2021;87:1338–46. 10.1111/bcp.14521 32803772

[175] Garg S, Sabri D, Kanji J et al. Evaluation of vitamin D medicines and dietary supplements and the physicochemical analysis of selected formulations. J Nutr Health Aging 2013;17:158–61. 10.1007/s12603-012-0090-423364495

[176] Verkaik-Kloosterman J, Seves SM, Ocké MC. Vitamin D concentrations in fortified foods and dietary supplements intended for infants: implications for vitamin D intake. Food Chem 2017;221:629–35. 10.1016/j.foodchem.2016.11.128 27979251

[177] Khadgawat R, Ramot R, Chacko KM et al. Disparity in cholecalciferol content of commercial preparations available in India. Indian J Endocrinol Metab 2013;17:1100–3. 10.4103/2230-8210.122638 24381892 PMC3872693

[178] LeBlanc ES, Perrin N, Johnson JD Jr et al. Over-the-counter and compounded vitamin D: is potency what we expect? JAMA Intern Med 2013;173:585–6. 10.1001/jamainternmed.2013.3812 23400578

[179] Nimalaratne C, Sun C, Wu J et al. Quantification of selected fat soluble vitamins and carotenoids in infant formula and dietary supplements using fast liquid chromatography coupled with tandem mass spectrometry. Food Res Int 2014;66:69–77.

[180] Huang Z, You T. Personalise vitamin D(3) using physiologically based pharmacokinetic modelling. CPT: Pharmacometrics Syst Pharmacol 2021;10:723–34. 10.1002/psp4.12640 33960722 PMC8302240

[181] Wan M, Green B, Iyengar AA et al. Population pharmacokinetics and dose optimisation of colecalciferol in paediatric patients with chronic kidney disease. Br J Clin Pharmacol 2022;88:1223–34. 10.1111/bcp.15064 34449087 PMC9291800

[182] de Oliveira LF, de Azevedo LG, da Mota Santana J et al. Obesity and overweight decreases the effect of vitamin D supplementation in adults: systematic review and meta-analysis of randomized controlled trials. Rev Endocr Metab Disord 2020;21:67–76. 10.1007/s11154-019-09527-7 31832878

[183] Shroff R, Aitkenhead H, Costa N et al. Normal 25-hydroxyvitamin D levels are associated with less proteinuria and attenuate renal failure progression in children with CKD. J Am Soc Nephrol 2016;27:314–22. 10.1681/ASN.2014090947 26069294 PMC4696567

[184] Aloni Y, Shany S, Chaimovitz C. Losses of 25-hydroxyvitamin D in peritoneal fluid: possible mechanism for bone disease in uremic patients treated with chronic ambulatory peritoneal dialysis. Miner Electrolyte Metab 1983;9:82–86. 6601753

[185] National Kidney Foundation . K/DOQI clinical practice guidelines for bone metabolism and disease in chronic kidney disease. Am J Kidney Dis 2003;42:S1–201. 14520607

[186] Nadeem S, Tangpricha V, Ziegler TR et al. Randomized trial of two maintenance doses of vitamin D in children with chronic kidney disease. Pediatr Nephrol 2022;37:415–22. 10.1007/s00467-021-05228-z 34392411

[187] Feng Z, Lu K, Ma Y et al. Effect of a high vs. standard dose of vitamin D3 supplementation on bone metabolism and kidney function in children with chronic kidney disease. Front Pediatr 2022;10:990724. 10.3389/fped.2022.990724 36405836 PMC9673817

[188] Nata N, Kanchanasinitth J, Tasanavipas P et al. Efficacy of weekly split versus single doses of ergocalciferol on serum 25-hydroxyvitamin D among patients on continuous ambulatory peritoneal dialysis: a randomized controlled trial. Int J Nephrol 2021;2021:1. 10.1155/2021/5521689 PMC7984910 33791128

[189] Bischoff-Ferrari HA, Dawson-Hughes B, Orav EJ et al. Monthly high-dose vitamin D treatment for the prevention of functional decline: a randomized clinical trial. JAMA Intern Med 2016;176:175–83. 10.1001/jamainternmed.2015.7148 26747333

[190] Sanders KM, Stuart AL, Williamson EJ et al. Annual high-dose oral vitamin D and falls and fractures in older women: a randomized controlled trial. JAMA 2010;303:1815–22. 10.1001/jama.2010.594 20460620

[191] Morrone L, Palmer SC, Saglimbene VM et al. Calcifediol supplementation in adults on hemodialysis: a randomized controlled trial. J Nephrol 2022;35:517–25. 10.1007/s40620-021-01104-z 34173940

[192] Donati S, Marini F, Giusti F et al. Calcifediol: why, when, how much? Pharmaceuticals (Basel) 2023; 6:637. 10.3390/ph16050637 PMC10222038 37242420

[193] Pérez-Castrillón JL, Dueñas-Laita A, Brandi ML et al. Calcifediol is superior to cholecalciferol in improving vitamin D status in postmenopausal women: a randomized trial. J Bone Miner Res 2020;36:1967–78. 10.1002/jbmr.4387 PMC8597097 34101900

[194] Cashman KD, Seamans KM, Lucey AJ et al. Relative effectiveness of oral 25-hydroxyvitamin D3 and vitamin D3 in raising wintertime serum 25-hydroxyvitamin D in older adults. Am J Clin Nutr 2012;95:1350–6. 10.3945/ajcn.111.031427 22552038

[195] Fadda G, Germain MJ, Broumand V et al. Real-world assessment: clinical effectiveness and safety of extended-release calcifediol. Am J Nephrol 2021;52:798–807. 10.1159/000518545 34818216

[196] Germain MJ, Paul SK, Fadda G et al. Real-world assessment: effectiveness and safety of extended-release calcifediol and other vitamin D therapies for secondary hyperparathyroidism in CKD patients. BMC Nephrology 2022;23:362. 10.1186/s12882-022-02993-3 36368937 PMC9650892

[197] Sempos CT, Durazo-Arvizu RA, Dawson-Hughes B et al. Is there a reverse J-shaped association between 25-hydroxyvitamin D and all-cause mortality? Results from the U.S. nationally representative NHANES. J Clin Endocrinol Metab 2013;98:3001–9. 10.1210/jc.2013-1333 23666975 PMC3701270

[198] Durup D, Jørgensen HL, Christensen J et al. A reverse J-shaped association of all-cause mortality with serum 25-hydroxyvitamin D in general practice: the CopD study. J Clin Endocrinol Metab 2012;97:2644–52. 10.1210/jc.2012-1176 22573406

[199] Durup D, Jørgensen HL, Christensen J et al. A reverse J-shaped association between serum 25-hydroxyvitamin D and cardiovascular disease mortality: the CopD study. J Clin Endocrinol Metab 2015;100:2339–46. 10.1210/jc.2014-4551 25710567

[200] Zhao S, Chen X, Wan Z et al. Associations of serum 25-hydroxyvitamin D and vitamin D receptor polymorphisms with risks of cardiovascular disease and mortality among patients with chronic kidney disease: a prospective study. Am J Clin Nutr 2024; 119:1397–404. 10.1016/j.ajcnut.2024.04.001 38608754

[201] Tantiyavarong P, Kramer A, Heaf JG et al. Changes in clinical indicators related to the transition from dialysis to kidney transplantation—data from the ERA-EDTA Registry. Clin Kidney J 2020;13:188–98. 10.1093/ckj/sfz062 32296524 PMC7147310

[202] Rolighed L, Rejnmark L, Sikjaer T et al. Vitamin D treatment in primary hyperparathyroidism: a randomized placebo controlled trial. J Clin Endocrinol Metab 2014;99:1072–80. 10.1210/jc.2013-3978 24423366

[203] Holick MF, Binkley NC, Bischoff-Ferrari HA et al. Evaluation, treatment, and prevention of vitamin D deficiency: an Endocrine Society clinical practice guideline. J Clin Endocrinol Metab 2011;96:1911–30. 10.1210/jc.2011-0385 21646368

[204] Lips P, Cashman KD, Lamberg-Allardt C et al. Current vitamin D status in European and Middle East countries and strategies to prevent vitamin D deficiency: a position statement of the European Calcified Tissue Society. Eur J Endocrinol 2019;180:P23–54. 10.1530/EJE-18-0736 30721133

[205] Kaur J, Khosla S, Farr JN. Effects of diabetes on osteocytes. Curr Opin Endocrinol Diabetes Obes 2022;29:310–7. 10.1097/MED.0000000000000733 35749726 PMC9271606

[206] Liyanage P, Lekamwasam S, Weerarathna TP et al. Effect of vitamin D therapy on urinary albumin excretion, renal functions, and plasma renin among patients with diabetic nephropathy: a randomized, double-blind clinical trial. J Postgrad Med 2018;64:10–15. 10.4103/jpgm.JPGM_598_16 29386413 PMC5820809

[207] Nata N, Siricheepchaiyan W, Supasyndh O et al. Efficacy of high versus conventional dose of ergocalciferol supplementation on serum 25-hydroxyvitamin D and interleukin-6 levels among hemodialysis patients with vitamin D deficiency: a multicenter, randomized, controlled study. Ther Apher Dial 2022;26:378–86. 10.1111/1744-9987.13722 34378863

[208] Mager DR, Jackson ST, Hoffmann MR et al. Vitamin D(3) supplementation, bone health and quality of life in adults with diabetes and chronic kidney disease: results of an open label randomized clinical trial. Clin Nutr 2017;36:686–96. 10.1016/j.clnu.2016.05.012 27302208

