# Supplementary material for: The role of nutritional vitamin D in chronic kidney disease–mineral and bone disorder in children and adults with chronic kidney disease, on dialysis, and after kidney transplantation—a European consensus statement
Source: Nephrol Dial Transplant. 2025 Jan 28;40(4):797–822. doi: 10.1093/ndt/gfae293 (PMC11960744; doi:10.1093/ndt/gfae293)
Supplement: gfae293_Supplemental_File [file gfae293_supplemental_file.pdf]

Supplementary tables

Table S1: Randomised trials investigating the effect of nutritional vitamin D supplementation on fibroblast growth factor 23 (FGF23) levels in patients with chronic kidney disease (divide by 2.5 to convert from nmol/L to ng/mL)

Study	n	Population	CKD grade	Supplement and dose	Follow-up	FGF23 levels as outcome
Kamath 2023<br><br>Iyengar 2022	90	Children <18 yrs	G3-4	Chol 3000 IU /d or<br>25000 IU /wk *or*<br>100000 IU /mo for 3 mo	3 mo	No significant differences between groups
Matuszkiewicz-Rowinska 2022	62	Adults	G5D (HD)	Chol 4000 IU x3/wk for 13 wks *vs placebo*	13 wks	No significant differences between groups
Westerberg 2018	95	Adults	G3-4	Chol 8000 IU /d for 12 wks *vs placebo*	12 wks	No significant differences between groups
Alvarez 2013	48	Adults	G2-3	Chol 50000 IU /wk for 12 wks, then 50000 IU /2wk for 40 wks *vs placebo*	12 & 52 wks	No significant differences between groups. In subgroup with baseline 25(OH)D ≥75 nmol/L, increased FGF23 at wk 12 which returned to baseline at wk 52.
Alshahawey 2021	60	Adults	G5D (HD)	Chol 200000 IU /mo for 3 mo *vs placebo*	3 mo	No significant differences between groups
Kumar 2017	120	Adults	G3-4	Chol 300000 IU at 0 and 8 wks v*s placebo*	16 wks	No significant differences between groups
Sprague 2016	429	Adults	G3-4	Calcifediol (extended release) 30 – 60 mcg /d *vs placebo*	26 wks	No significant differences between groups at any time point (measured every 2 or 4 weeks).
Sprague 2015	78	Adults	G3-4	Calcifediol (extended release) 30, 60, or 90 mcg /d for 6 wks *vs placebo*	6 wks	No significant differences between groups

Abbreviations: 25(OH)D = 25-hydroxyvitamin-D; Chol=Cholecalciferol; FGF23 = fibroblast growth factor 23; IU = International unit; mcg = microgram

Table S2: Systematic reviews and meta-analyses investigating different types of nutritional vitamin D in the general population (divide by 2.5 to convert from nmol/L to ng/mL)

**Study**	**Population**	**No. of studies**	**Interventions**	**Conclusion**
van den Heuvel 2024	Healthy adults	17	Ergocalciferol vs. Cholecalciferol as daily or x1-2 wk dosing	Cholecalciferol lead to a greater increase of 25(OH)D than ergocalciferol, even if limited to daily dose studies. Based on 12 studies using LC-MS/MS, the mean difference was 10 nmol/l lower for ergocalciferol (95% CI: −15 to −6).
Balachandar 2021	Apparently healthy – all ages	25	Ergocalciferol vs. Cholecalciferol	Cholecalciferol showed higher efficacy in improving 25(OH)D (mean difference: 16 nmol/l, 95% CI: 9 to 22) and reducing PTH levels (mean difference: −0.6 pmol/l; 95% CI: −0.9 to −0.2), across variable participant demographics, dosage, and vehicle of supplementation.
Tripkovic 2012	Healthy adults	10	Ergocalciferol vs. Cholecalciferol	Cholecalciferol was more efficacious at raising 25(OH)D concentrations than ergocalciferol, but the effect was lost with daily supplementation.

Abbreviations: 25(OH)D = 25-hydroxyvitamin-D; CI = Confidence interval; HD = Haemodialysis; LC-MS/MS = Liquid chromatography-tandem mass spectrometry; PTH = Parathyroid hormone

Table S3: Randomised trials comparing different routes of administration of nutritional vitamin D in adult patients with chronic kidney disease (divide by 2.5 to convert from nmol/L to ng/mL)

**Study**	**n**	**CKD stage**	**Baseline 25(OH)D** (nmol/l)	**Oral intervention**	**Intramuscular intervention**	**Outcome**
Behairy 2022[158]	80	G5D (HD)	Oral: 30<br>IM: 26	Cholecalciferol 25000 IU /wk for 12 wks	Cholecalciferol 300000 IU as a single dose	Intramuscular cholecalciferol is more effective at increasing 25(OH)D and reducing PTH.

Abbreviations: 25(OH)D = 25-hydroxyvitamin-D; HD = Haemodialysis; IM = Intramuscular; IU = International unit; PTH = Parathyroid hormone

Table S4: Studies assessing percentage deviation between measured vitamin D content and labelled value of vitamin D containing products.

**Study**	**Country**	**Products tested**, N	**% deviation between measured and labelled value**	**Products where % deviation falls between 90% to 110%**, n (%)
Products considered to be pharmaceutical-grade in their respective countries				
Wan 2021	England	2	91 – 91%	2 (100)
Garg 2013	New Zealand	2	90 – 97%	2 (100)
Khadgawat 2013	India	14	9 – 165%	2 (14)
Over-the-counter dietary supplements				
Wan 2021	England	11	41 – 165%	1 (9)
Verkaik-Kloosterman 2017	Netherlands	10	8 – 177%	Not reported
Nimalaratne 2014	Canada	4	66 – 145%	1 (25)
Garg 2013	New Zealand	12	8 – 201%	6 (50)
LeBlanc 2013	USA	15	52 – 135%	7 (47)

Table S5: Randomised trials of nutritional vitamin D comparing different doses in children with chronic kidney disease (divide by 2.5 to convert from nmol/L to ng/mL)

**Study**	**N**	**CKD stage**	**Baseline 25(OH)D** (nmol/l)	**Vitamin D compound**	**Arm 1**	**Arm 2**	**Follow-up**	**Outcome**
Nadeem 2022	98	G3-5	Arm 1: 65<br>Arm 2: 68	Chol	4000 IU /d	1000 IU /d	6 mo	74% (CI: 59; 87%) patients in the higher dose group had 25(OH)D >75 nmol/L at 6 mo vs 33% (CI: 18; 52%) in the lower dose group.
Feng 2022	150	G2-5	Arm 1: 13<br>Arm 2: 14	Chol	2000 IU /d	400 IU /d	4 mo	Greater increase of 25(OH)D in the high-dose group (+2.3 nmol/L) vs the low-dose (+1.1 nmol/L), but 25(OH)D levels are sub-therapeutic in both groups.

Abbreviations: 25(OH)D = 25-hydroxyvitamin-D; Chol=Cholecalciferol

Table S6: Randomised controlled trials of intermittent or single high-dose nutritional vitamin D on risk of falls

Study	n	Population	Mean age (year)	Vitamin D compound	Route	Intervention	Control	Duration of therapy	Relative risk of falls (95% CI)
Bischoff-ferrari 2016	200	Community-dwelling people with a prior fall	77.7	Chol	Oral	60000 IU /mo or 24,000 IU /mo (+ 300 mcg calcifediol)	Placebo	1 yr	1.40 (1.06, 1.85)
Waterhouse 2021	2200	General population	72.1	Chol	Oral	60000 IU /mo	Placebo	4.9 yrs	1.04 (0.85, 1.28)
Ginde 2017	107	Care home residents	81	Chol	Oral	100000 IU /mo	Placebo (or 12,000 IU as usual care)	1 yr	1.26 (0.73, 2.19)
Khaw 2017	5108	Healthy	65.9	Chol	Oral	100000 IU /mo	Placebo	3.4 yrs	0.98 (0.93, 1.03)
Law 2006	3717	People in residential accommodation	85	Ergo	Oral	100000 IU /3 mo	No treatment	7– 4 mo	1.03 (0.95, 1.10)
Glendenning 2011	686	Postmenopausal women	70	Chol	Oral	150000 IU /3 mo (+ Ca)	Placebo (+ calcium)	9 mo	1.08 (0.85, 1.38)
Latham 2003	243	Frail older people	79.5	Chol	Oral	300000 IU single dose	Placebo	Single dose	1.13 (0.89, 1.42)
Harwood 2004	150	Women following surgery for hip fracture	81.2	Ergo	IM	300000 IU single dose (+/- Ca) or 800 IU/d oral Chol (+ Ca)	No treatment	Single dose	0.48 (0.26, 0.90)
Smith 2007	9440	Elderly	75+	Ergo	IM	300000 IU /yr	Placebo	3 yrs	0.98 (0.98, 1.02)
Sanders 2010	2256	Women	70+	Chol	Oral	500000 IU /yr	Placebo	3 – 5 yrs	1.08 (1.03, 1.14)
Dhesi 2004	139	Patients attending a falls clinic	76.8	Ergo	IM	600000 IU single dose	Placebo	Single dose	0.77 (0.38, 1.59)

References and relative risk data from Myung et al., 2023. Abbreviations: 25(OH)D = 25-hydroxyvitamin-D; Chol=Cholecalciferol; Ergo=Ergocalciferol; IM = Intramuscular; IU = International unit

Table S7: Results of the Delphi survey

		Strongly agree	Agree	Neutral	Disagree	Strongly disagree	*Overall agree*	*Overall disagree*
2.2	In patients with CKD G3-5D, we suggest to measure 25(OH)D at first presentation, with repeated analysis 3 months after a 25(OH)D targeting intervention (e.g. change of supplementation dose or interval), and at least annually.	40%	49%	6%	4%	1%	89%	6%
2.2	Routine monitoring of 1,25(OH)2D levels, free vitamin D levels, and ratios between vitamin D metabolites are not recommended in patients with CKD.	33%	47%	13%	6%	1%	80%	7%
3.1	We recommend supplementing vitamin D to >75 nmol/L (>30 ng/mL) in adults and children with CKD G2-5D to delay the onset of and improve the control of secondary hyperparathyroidism.	46%	47%	6%	1%	0%	93%	1%
3.4	We suggest to supplement vitamin D to >75 nmol/L (>30 ng/mL) in kidney transplant recipients to reduce the risk of bone loss and fractures post-transplant.	37%	51%	10%	1%	0%	89%	1%
4.1	We suggest supplementing vitamin D for cardiovascular risk mitigation only in selected patients with severe vitamin D deficiency.	24%	40%	27%	6%	3%	64%	9%
5.1	We suggest using oral cholecalciferol to increase and maintain serum 25(OH)D concentration in the target range. If cholecalciferol is unavailable, oral ergocalciferol may be used in an equivalent dose.	34%	51%	14%	0%	0%	86%	0%
5.1	Oral administration of cholecalciferol may be considered for most patients, with intramuscular administration reserved for people with gastrointestinal malabsorption disorders.	39%	50%	7%	4%	0%	89%	4%
5.1	Administer vitamin D compounds by mouth and not through feeding tubes.	27%	39%	33%	1%	0%	66%	1%

5.1	We suggest replenishing vitamin D before using an active vitamin D compound in CKD G1-3 and after kidney transplantation, and before or concomitantly with an active vitamin D compound in CKD G4-5D.	39%	51%	9%	1%	0%	90%	1%
5.2	We suggest using oral cholecalciferol with once daily, weekly, fortnightly, or monthly dosing schedules, adjusting the dose for baseline 25(OH)D levels and body size.	31%	54%	11%	3%	0%	86%	3%
5.2	In adults with CKD G2-5D or after kidney transplantation with serum 25(OH)D concentration <75 nmol/L (<30 ng/mL), we suggest an equivalent daily dose of 5,000 – 7,000 IU/day of cholecalciferol for a duration of 12 weeks to achieve the optimal target range. To maintain serum 25(OH)D concentration in the target range, we suggest continuous dosing with an equivalent daily dose of 2,000 IU/day.	23%	59%	13%	6%	0%	81%	6%
5.2	In children with CKD G2-5D or after kidney transplantation with serum 25(OH)D concentration <75 nmol/L (<30 ng/ml), we suggest an equivalent daily dose of 3,000-7,000 IU/day of cholecalciferol for a duration of 12 weeks to achieve the optimal target range. To maintain serum 25(OH)D concentration in the target range, we suggest continuous dosing with an equivalent daily dose of 1,000-2,000 IU/day, adjusting for body size.	21%	50%	24%	4%	0%	71%	4%
5.2	We suggest to avoid exceeding doses of 100,000 IU given as a single dose.	61%	24%	10%	3%	1%	86%	4%
5.3	Calcifediol, where available, may be considered instead of cholecalciferol or ergocalciferol in adults with CKD G3-4 or liver failure.	17%	49%	27%	7%	0%	66%	7%
5.4	In adults and children with CKD or after kidney transplantation, we suggest withholding nutritional vitamin D supplements when serum 25(OH)D levels are above 150-200 nmol/L (60-80 ng/ml), in the absence of hypercalcaemia.	34%	53%	9%	4%	0%	87%	4%

5.4	In patients with CKD and hypercalcaemia check for and manage other causes of high calcium including iatrogenic (the use of active vitamin D compounds, high dialysate calcium, oral calcium-containing medications), CKD-related (presence of tertiary hyperparathyroidism), and non-CKD related conditions (malignancy, haematological conditions, sarcoidosis, etc) before stopping cholecalciferol supplementation.	39%	46%	6%	9%	1%	84%	10%

Note: A Delphi panel of adult and pediatric nephrologists with an interest in chronic kidney disease – mineral and bone disorder was asked to review the manuscript draft and rate the clinical practice points on a 5-point scale from 'strongly agree' to 'strongly disagree'.
An overall agreement ('Strongly agree' + 'Agree') >70% was the predefined goal.
